# Synthesis, kinetic studies and *in-silico* investigations of novel quinolinyl-iminothiazolines as alkaline phosphatase inhibitors

**DOI:** 10.1080/14756366.2022.2163394

**Published:** 2023-01-11

**Authors:** Muhammad Naeem Mustafa, Pervaiz Ali Channar, Muhammad Sarfraz, Aamer Saeed, Syeda Abida Ejaz, Mubashir Aziz, Fatmah Ali Alasmary, Hanadi Yaqob Alsoqair, Hussain Raza, Song Ja Kim, Asad Hamad

**Affiliations:** a Department of Chemistry, Quaid-i-Azam University, Islamabad, Pakistan; b Department of Basic sciences and Humanities, Dawood University of Engineering and Technology, Karachi, Pakistan; c College of Pharmacy, Al Ain Campus, Al Ain University, Al Ain, United Arab Emirates; d Department of Pharmaceutical Chemistry, The Islamia University of Bahawalpur, Bahawalpur, Pakistan; e Department of Chemistry, College of Science, King Saud University, Riyadh, Saudi Arabia; f Department of Biological Sciences, College of Natural Sciences, Kongju National University, Gongju, Republic of Korea; g Faculty of Pharmacy, Grand Asian University Sialkot, Sialkot, Pakistan

**Keywords:** Alkaline phosphatase, synthesis, DFT, molecular docking, kinetic analysis

## Abstract

Deposition of hydroxyapatite (HA) or alkaline phosphate crystals on soft tissues causes the pathological calcification diseases comprising of end-stage osteoarthritis (OA), ankylosing spondylitis (AS), medial artery calcification and tumour calcification. The pathological calcification is symbolised by increased concentration of tissue non-specific alkaline phosphatase (TNAP). An efficient therapeutic strategy to eradicate these diseases is required, and for this the alkaline phosphatase inhibitors can play a potential role. In this context a series of novel quinolinyl iminothiazolines was synthesised and evaluated for alkaline phosphatase inhibition potential. All the compounds were subjected to DFT studies where *N*-benzamide quinolinyl iminothiazoline (**6g**), *N*-dichlorobenzamide quinolinyl iminothiazoline (**6i**) and *N*-nitrobenzamide quinolinyl iminothiazoline (**6j**) were found as the most reactive compounds. Then during the *in-vitro* testing, the compound *N*-benzamide quinolinyl iminothiazoline (**6g**) exhibited the maximum alkaline phosphatase inhibitory effect (IC_50_ = 0.337 ± 0.015 µM) as compared to other analogues and standard KH_2_PO_4_ (IC_50_ = 5.245 ± 0.477 µM). The results were supported by the molecular docking studies, molecular dynamics simulations and kinetic analysis which also revealed the inhibitory potential of compound *N*-benzamide quinolinyl iminothiazoline (**6g**) against alkaline phosphatase. This compound can be act as lead molecule for the synthesis of more effective inhibitors and can be suggested to test at the molecular level.

## Graphical abstract

**Figure:**
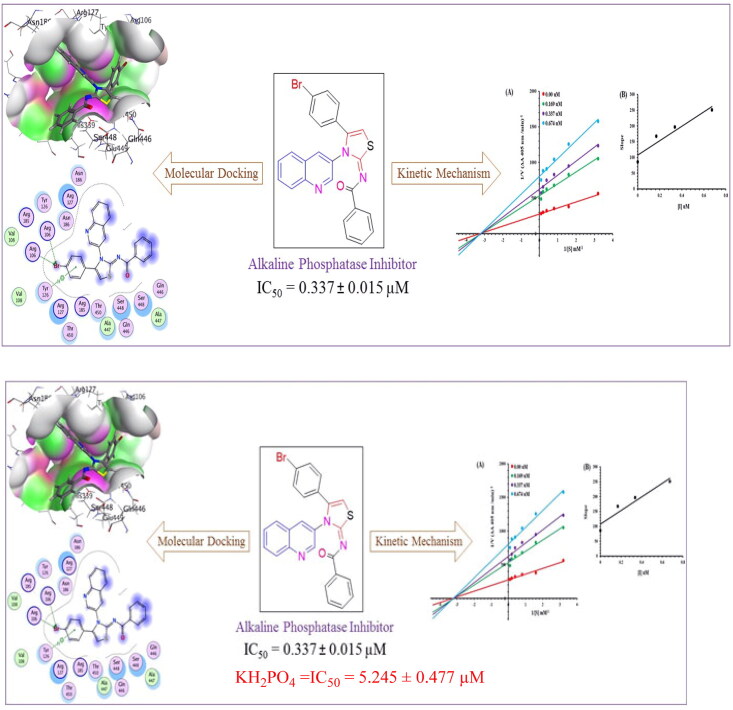

## Introduction

Alkaline phosphatases (APs) are metalloenzymes that contain nucleotide metabolising enzymes^1^. These APs are abundantly found in nature, including humans and bacteria^2^. These enzymes play a crucial role in catalysing the hydrolytic dephosphorylation of nucleotides into nucleosides^3^. Moreover, they are significantly involved in various signalling pathways, including cell signalling via the production of nucleoside from adenine monophosphate (AMP)^4^, the maturation pathways of adipocytes and osteoblasts^5^, and purinergic cell signalling pathway^6^. These are important players in the adipogenesis process^7^. Tissue non-specific alkaline phosphatase (TNAP) is one of four types of APs, and the other three are tissue-specific enzymes, including germ cell alkaline phosphatase (GCAP), placental alkaline phosphatase (PLAP), and intestinal alkaline phosphatase (IAP)^8^. The IAP is localised to the duodenum, a part of the gastrointestinal tract, and is an important drug target for inflammatory bowel disease and drug-associated diarrhea^9^. The overexpression of TNAP is associated with different diseases including various cancers and associated neurodegenerative diseases and complications^10^. Conclusively, aberrant expression of serum AP levels could result in severe malignancies and bone disorders. Furthermore, the enhanced concentration of alkaline phosphatase in tissues causes calcific diseases^11^ and these calcific diseases have effects on both skeletal (joints and bones) and non-skeletal tissues^2^. It also include ankylosing spondylitis (AS) in ligaments or tendons, pathological calcifications causing end-stage osteoarthritis (OA) in joint cartilage, medial artery calcification in the tunica media, or tumour calcification (i.e. in breast cancer)^12^. These diseases are relatively complex and multi-factorial as most of them are age associated diseases. Since most of these diseases promote inflammatory responses, the administration of anti-inflammatory drugs has been considered effective strategy to relieve pain in patients. The healthcare management has recommended anti-TNF drugs like adalimumab^13^, etanercept^14^ and infliximab^15^^,^^16^ for better quality of patients’ life. Although anti-inflammatory drugs are being used to reduce pain but these drugs do not cure and reduce calcification for patients. Now a days, attention has been dedicated to the development of drugs directly targeting the calcification process^17^^,^^18^. Calcification is accelerated by augmented expression of tissue non-specific alkaline phosphatase (TNAP) in cells with mineral competency. While the TNAP inhibitors reduce the calcification process and hence diseases associated with calcification can be avoided^17^.

**Figure 1.**
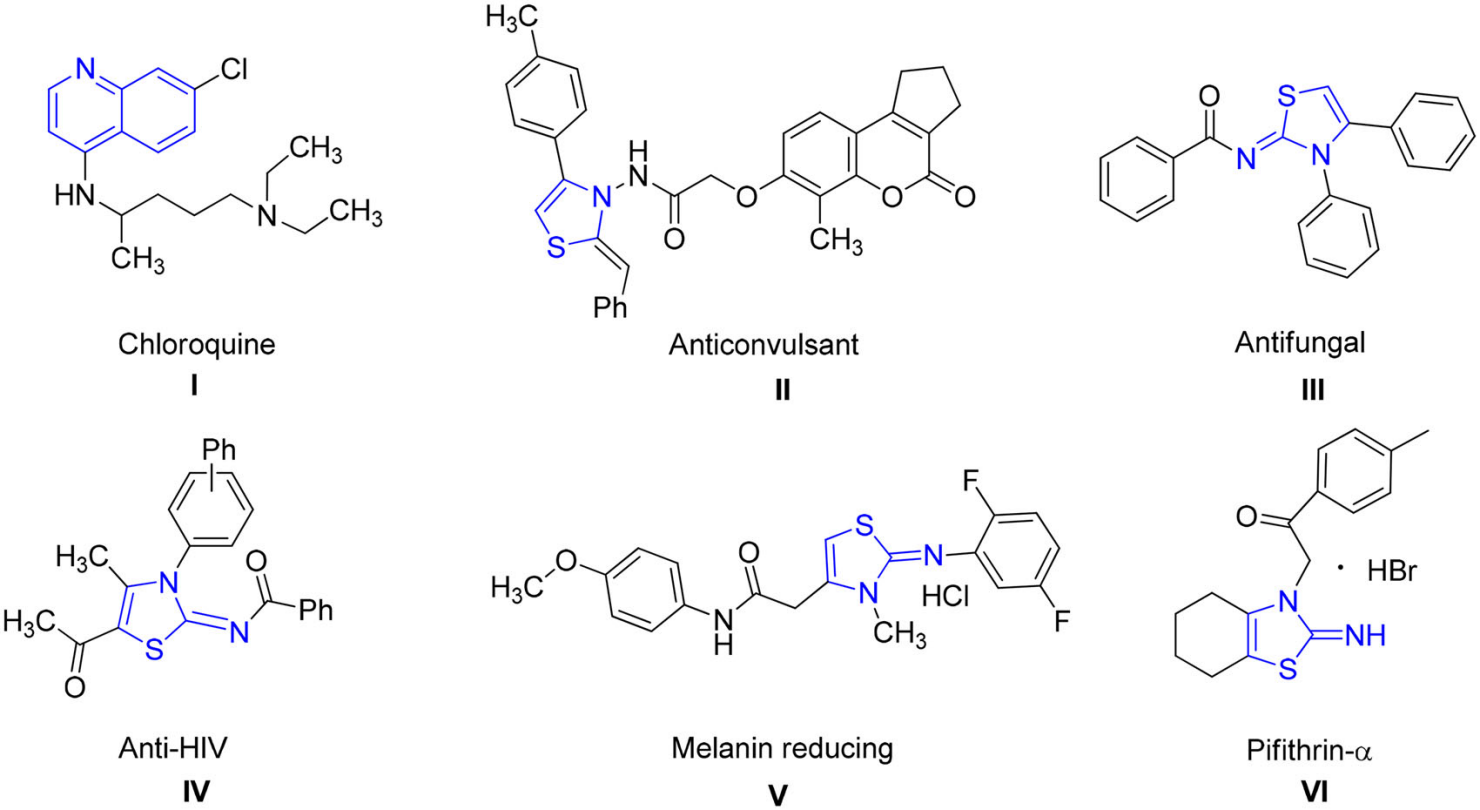
Structures of biologically active quinoline and thiazolines.

In the quest for safe drug therapy, researchers are striving for development of novel and specific medication for APs associated malignancies. Among various heterocyclic compounds, the quinolone and thiazolines possessed a wide range of biological and pharmacological activities including anti-cancer, anti-fungal, analgesic, antihypertensive and antibiotic activities. Specifically, quinoline has displayed an broad spectrum of biological applications such as anti-fungal, antimalarial, anticonvulsant, analgesic, anti-bacterial, cardiotonic anthelmintic, and anti-inflammatory activities^19^. Several pharmacologically active substances and natural products (Cinchona Alkaloids) possess quinoline nucleus^20^. The most famous quinoline based drug chloroquine **I** (Figure 1) resulted in eradication and control of malaria for the decades. This type of drugs influence parasite’s life cycle during blood stages^21^.

The second class of compounds i.e. thiazoline moiety also possesses various pharmacological and therapeutic applications in the drug industries. These derivatives exhibit anti-allergic^22^, antibiotic^23^, anticonvulsant **II**^24^^,^^25^, antifungal **III**^26^, antihypertensive^27^, anti-HIV **IV**^28^, anti-inflammatory^28^, antimalarial, antipyretic, antirheumatic, antitumor^29^, analgesic^30^ and cytotoxic activities^31^. 3-Methylthiazolidine is appreciated for the inhibition of indole ethylamine *N*-methyltransferase (INMT)^32^ and beneficial for treatment of schizophrenia^33^. Bayer CropScience developed commercially available iminothiazoline insecticide Thiacloprid^34^. Moreover, 2-imino-3-(benzoylmethyl)thiazolidine is used to protect from γ-radiation^35^. Likewise, hydroxy thiazole carboxylate II was stated for the inhibition of HIF-*α* prolyl hydroxylase while PS-028 is selective GPIIb/IIIa antagonist^36^^,^^37^. Significantly, 2-Imino-1,3-thiazoline scaffolds **V** impede production of melanin in dose-dependent style, thus acting as whitening agent of skin^38^. Pifithrin-α **VI** is recognised iminothiazoline reversible inhibitor of p53-dependant gene transcription and p53-mediated apoptosis^39^. Thiazolines exhibit interesting applications such as insecticides, acaricides, and plant growth regulators in agriculture^40^. It also found in the natural products like mirabazoles, tantazoles and thiangazole, which show anti-HIV and anticancer activities^41^.

The compounds with iminothiazoline moiety also exhibited alkaline phosphatase inhibition activity^41^. In accordance with the significance of quinoline and iminothiazoline structural components, we have designed and synthesised quinoline based iminothiazoline with multiple point structural diversity to be evaluated reactive molecules with effective alkaline phosphatase inhibition potential which was supported by molecular docking and kinetic studies.

## Experimental

### General methodology

Utilising silica gel plates with pre-applied aluminium coatings, the compounds’ Rf-values were calculated. Using the open capillary method and the Gallenkamp melting point equipment, the melting points were calculated (MP-D). The IR analysis was carried out using the Bruker FT-IR Bio–Rad-Excalibur Series Mode No. FTS 300 MX spectrometer. When recording the ^1^H NMR and ^13^C NMR spectra with a Bruker 300 MHz NMR spectrometer in deuterated DMSO and CDCl_3_ solutions, tetra-methyl silane (TMS) was employed as an internal reference. For HPLC-MS analysis, an Agilent 1200 series LC system was employed, and elemental analyses were conducted using an LECO-183 CHNS analyser.

### General method for the synthesis of (E)-N-(4-(4-bromophenyl)-3-(quinolin-3-yl)thiazol-2(3H)-ylidene)alkyl/arylamide 6(a–j)

Potassium thiocyanate (1 mmol) was added in dry acetone (15 ml) in two necks round bottom flask fitted with reflux condenser and stirred for 5 min. The acetone solution of suitably substituted acid chlorides (1 mmol) was added dropwise to dissolve the potassium thiocyanate with stirring. The resulting mixture was heated to reflux the temperature for time of 3–4 h to afford the isothiocyanate. After cooling, an acetone solution of 3-aminoquinoline (1 mmol) was introduced dropwise and temperature of resulting solution was kept 60 °C for 12–14 h to afford the acyl thioureas on addition with ice cooled water. The resultant product was purified by recrystallization in acetone or ethanol.

In the round-bottom flask with 15 ml of dry dichloromethane and triethyl amine, acyl thioureas (1 mmol) was added (1 mmol). With an addition funnel, *p*-bromophenacyl bromide (1 mmol) solution in dichloromethane was dropped into this mixture over the course of 30 min. To produce the quinolinyl-iminothiazolines **6(a–j)**, the reaction mixture was heated to 50 °C for 24 h under a nitrogen environment. The solution was filtered, and solvent was extracted using a rotary evaporator when the reaction was completed. The completion of reaction was determined by thin layer chromatography (TLC). The solid products underwent recrystallization in ethanol for purification. The supplementary file contains the comprehensive characterisation information.

### Density functional theory calculations (DFT)

The structural geometries of synthesised derivatives were optimised and frequency calculations^42^ were performed using Gaussian 09W program^43^. Initially, structures were prepared in required format of sybyl mol2 format using Chem 3D Pro^44^^,^^45^. The files were loaded into the system and calculations were performed using B3LYP functional correlation and STO-3G basis set^46^. STO-3G basis set belongs to *slater* and *Gaussian type orbitals* with enhanced number of functions which ensure rapid convergence and ensure reduced computational cost. These sets are often used to produce accurate assumptions on mono and diatomic systems^47^. It is single zeta type basis set^48^ which provide *1s* function for first row elements, and two *2s* and one *p*(px, py, pz) function for second and third row elements [2s1p]^49–51^. STO-3G basis set is widely used for many elements of periodic table with good precision. The analysed structures were evaluated for frontier molecular orbitals^52^, HOMO/LUMO energy gap, chemical hardness and softness of derivatives^53^. The optimised files were visualised using Gauss View 6.0^54^.

### Alkaline phosphatase inhibition assay and kinetic mechanism analysis

The inhibitory activity of calf intestinal alkaline phosphatase was evaluated using spectrophotometric assay as previously reported in our studies^55^^,^^56^. In addition, Kinetic mechanism analysis was conducted to determine the mechanism of inhibition. The most potent molecule was selected on the basis of IC_50_ in order investigate the competitive or non-competitive enzyme inhibition by following our previously reported method^57^. The detailed procedure for alkaline phosphate inhibition assay and kinetic mechanism analysis is given in supplementary file.

### Molecular docking

The molecular docking procedure was then carried out using the optimised structures derived from DFT investigations. For the purpose of predicting interactions of derivatives inside the protein’s active pocket, the Molecular Operating Environment (MOE) 2015.10 was used^58^.

Initially, 3 dimensional crystallographic structure of alkaline phosphatase was retrieved form protein data bank (www.rcsb.com; PDB ID: 1alk). After retrieving 3D crystallographic structure of target, preparation of protein is important step to proceed for molecular docking. To begin, protein was dealt with the energy minimisation stage. Then, the atomic charges must always be adjusted, followed by the adaptation of the potential energy. Additionally, crucial characteristics were regulated using the MMFF94x force field including incorporation of polar hydrogens and removal of hetero atoms^59^. Second, the site finder was implemented to the protein structure, followed by the creation of spheres at selected residues of active pocket. Finally, the ligand library was exported in necessary format (MDB) and docked at selected residues. Total 100 poses were selected to represent the complex’s most stable configuration. To rank interaction efficiency, the scoring energy values were computed using the London dG scoring function, which was refined twice using triangular Matcher methods. Additionally, critical interaction data such as ligand receptor interactions and the amino-acid backbone involved, binding energy and kind of interaction were saved when the process was completed^60^. The docking protocol was validated by redocking co-crystal ligand and RMSD of produced pose was compared to native pose of the ligand. The RMSD value less than 2 angstroms validated the docking protocol. The visualisation of 3D and 2D poses of tope ranked conformation was retrieved form PyMOL^61^ and LigPlot plus^62^ respectively.

### Molecular dynamics (MD) simulations

Molecular dynamics simulation is inevitable to integrate the motion equation of atoms with respect to the reference frame^63^. Desmond software was used to simulate the protein–ligand complex for 100 ns in the TIP3P solvent model. The optimised potential for liquid simulations (OPLS3) forcefield^64^ was used to simulate the complex under PBC (periodic boundary conditions). The orthorhombic solvation box was used to solvate the system with TIP3P water molecules. The counter ions (NaCl) were incorporated at a concentration of 0.15 M for neutralisation purposes. The initial energy minimisation was carried out for 2000 steps using the steepest descent method to omit any steric clashes. At 300 K and 1.01 bar pressure, the system was adjusted in an isothermal and isobaric (NPT) ensemble. A cut-off distance of 10 angstroms was used to take into account short-range van der Waals interactions. A Martyna–Tobias–Klein barostat^65^ and Nose–Hoover thermostat were also utilised to keep the pressure steady and temperature during simulation^66^. The final production run was conducted for 100 ns, and trajectories were saved at every 100 ps. The time step of 2 fs was utilised to integrate the motion equations. The particle mesh Ewald method^67^ was employed for precise and reliable investigation of electrostatic interactions. The Desmond simulation interaction diagram protocol was used to analyse the simulated trajectories of protein–ligand complexes^68^.

## Results and discussion

### Chemistry

**Scheme 1.**
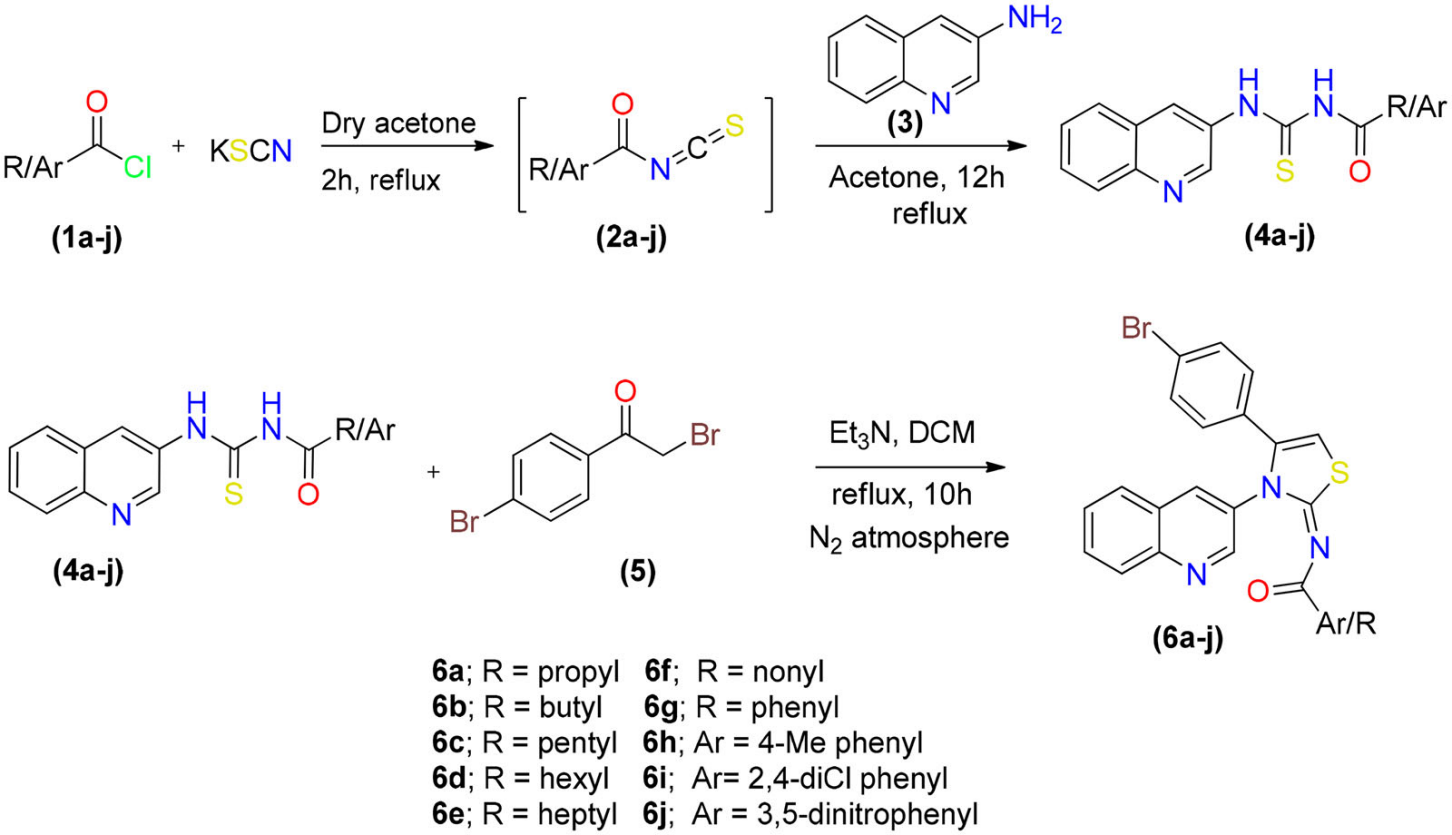
Synthetic route for synthesis of quinolinyl iminothiazoline **6(a–j)**.

By reacting potassium thiocyanate with various acid chlorides in dry acetone, followed by the addition of 3-aminoquinoline to produce the appropriate acyl thioureas, a range of new quinolinyl iminothiazolines were synthesised. After purification, the acyl thioureas were reacted with *p*-bromophenacylbromide to obtain the (*E*)-*N*-(4-(4-bromophenyl)-3-(quinolin-3-yl)thiazol-2(3*H*)-ylidene)alkyl/aryl amides **6**(**a–j)** as shown in Scheme 1. The reactions were carried out in dry solvents because intermediate **2** undergo hydrolysis in the presence of moisture. The last step was completed in inert atmosphere to avoid the formation of side products.

### Spectroscopic characterisation

The newly developed scaffolds of quinolinyl iminothiazolines were characterised by NMR, HPLC-MS and FT-IR. FT-IR spectra of synthesised compounds demostrated the absorption band for C–H aromatic at 3059–3115 cm^−1^, C–H thiazoline at 2957–3062 cm^−1^ and C = O at 1679–1738 cm^−1^. The ^1^H NMR spectra of compound **6(a–j)** contained three characteristic signals, aromatic protons of quinolinyl and phenyl ring appeared at *δ* 8.95–7.28 ppm, singlet of proton located at thiazoline ring observed at *δ* 6.39–6.53 ppm and alkyl protons give rise to signals at *δ* 2.37–0.86 ppm. In ^13^C NMR spectra signal for carbonyl carbon appeared at *δ* 171.2–173.6 ppm, signal for imine carbon of thiazoline ring observed at *δ* 168.9–171.3 ppm and carbon at 5-position of thiazoline ring give rise to signal at *δ* 103.6 − 106.2 ppm. Aromatic carbons of quinolinyl and phenyl moiety signals appeared at *δ* 149.4–126.4 ppm while aliphatic carbons were appeared at *δ* 30.5–13.5 ppm. These signals indicated the formation of desired analogue. HPLC plot of compound **6a** contained the band with retention time of 8.7 min. DAD spectrum involved two main band and one shoulder band. Among the two main bands, the band appeared at 343 nm was due to the quinolinyl group while the band observed at 237 nm was due to styrenyl moiety. The one shoulder band appeared at 255 nm was due to iminothiazoline ring with less conjugation. The positive mode ESI/MS of compound **6a** contained a peak at *m*/*z* = 453 assigned as molecular ion peak [M + H]^+^.

### Density functional theory studies

The functional correlation DFT/B3LYP was used to optimise the structures and geometry with the basis set STO-3G. Optimisation was the arranging of atoms in a molecule with the goal of minimising energy consumption. Table 1 summarises the optimised geometry parameters, including optimisation energy, polarizability, and dipole moment.

**Table 1.** Geometrical Parameters of selected compounds.

Sr. no.	Compound code	Optimisation energy (hartree)	Polarizability (α) (a.u.)	Dipole moment (Debye)
01.	**6a**	−4014.881	206.169	1.245
02.	**6b**	−4053.730	212.856	1.251
03.	**6c**	−4092.578	219.034	1.269
04.	**6d**	−4131.426	225.013	1.251
05.	**6e**	−4170.274	230.332	1.345
06.	**6f**	−4247.970	242.821	1.249
07.	**6g**	−4126.566	245.221	1.212
08.	**6h**	−4165.418	246.059	1.159
09.	**6i**	−5035.731	250.268	3.576
10.	**6j**	−4529.915	265.240	4.745

**Figure 2.**
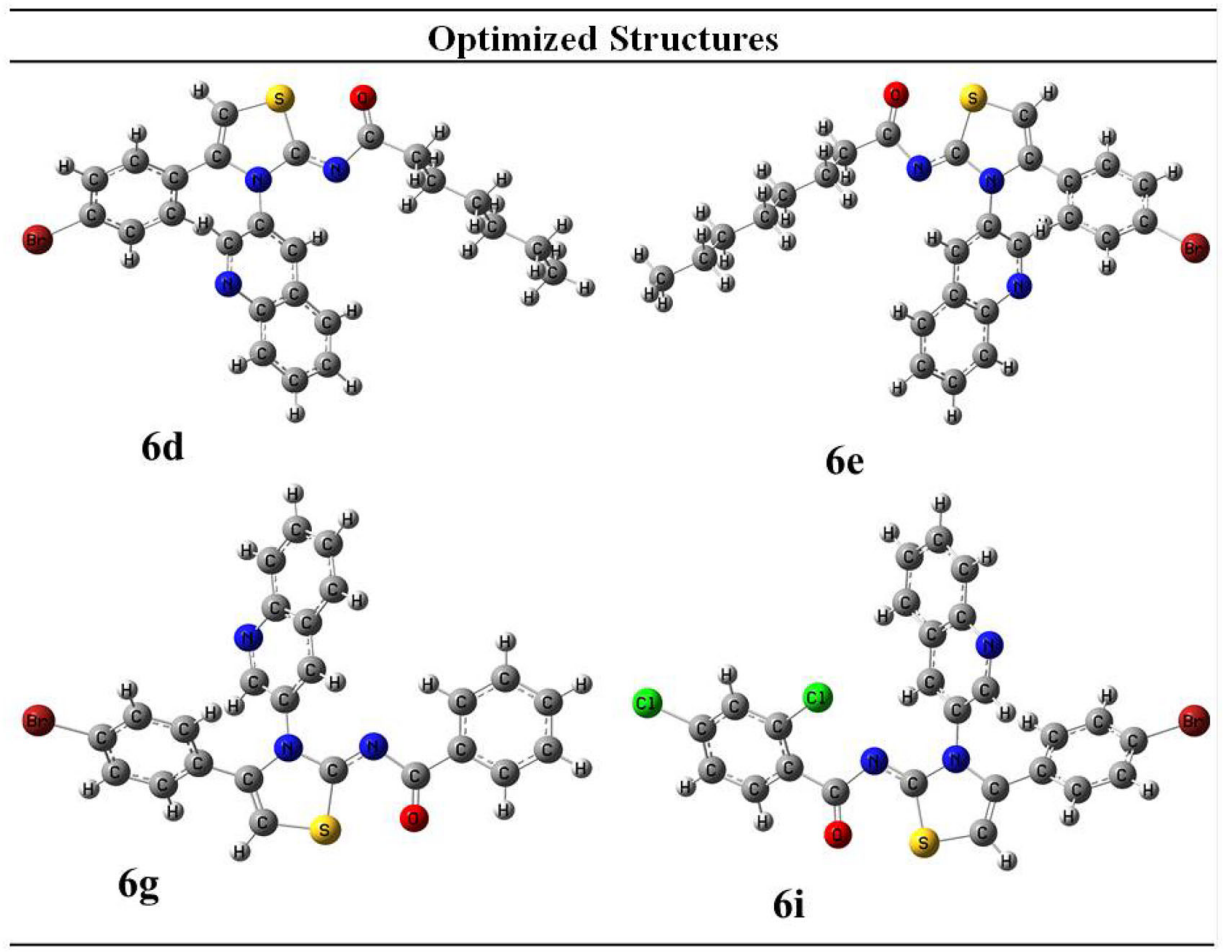
Optimised structures of potent compounds (**6d**, **6e**, **6g** and **6i**).

The synthesised derivatives’ obtained optimised geometries represented real local minima without the inclusion of imaginary frequencies. To achieve the sharpest energy gradient, these structural geometries were optimised. Figure 2 illustrates the optimised structures for several derivatives.

**Figure 3.**
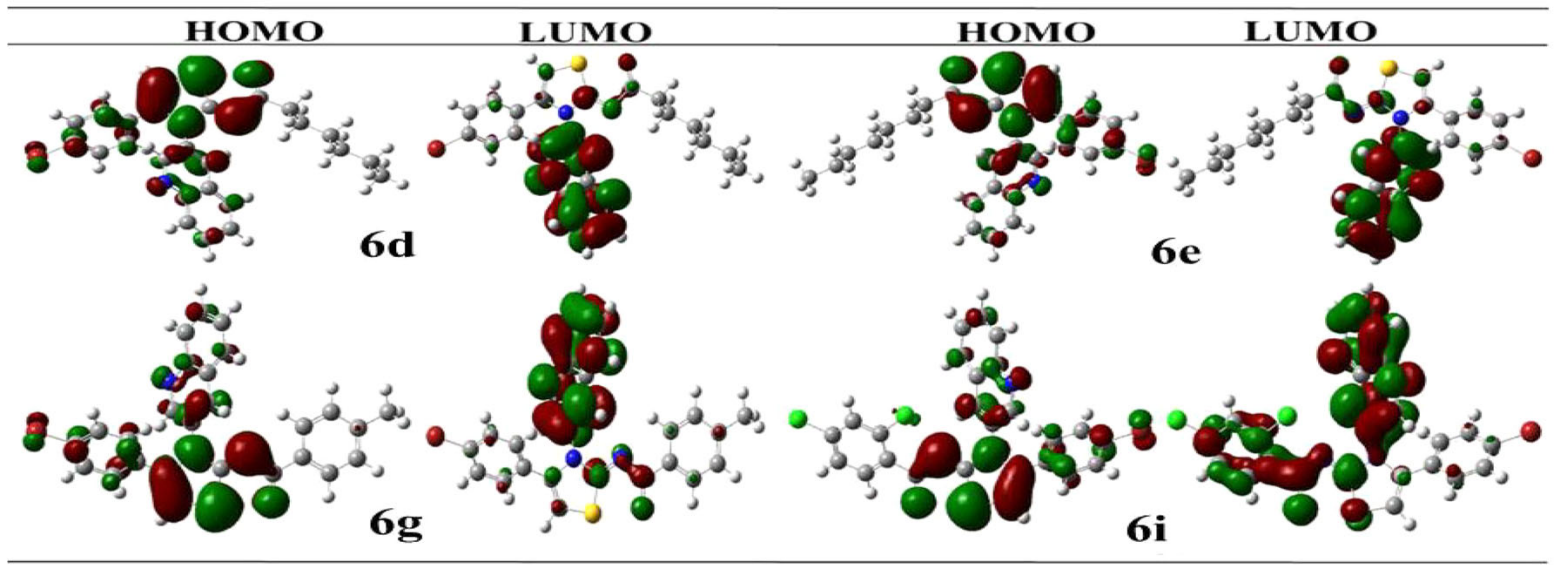
HOMO-LUMO structures of potent compounds (**6d**, **6e**, **6g** and **6i**).

The highest occupied molecular orbital, HOMO, possesses a nucleophilic characteristic. The lowest unoccupied molecular orbital, LUMO, is having electrophilic characteristic. The LUMO HOMO energy gap is critical for anticipating a compound’s reactivity. The majority of electronic changes have occurred in the LUMO HOMO gap. Because it is the outermost orbital, HOMO tends to give an electron. Due to the fact that the innermost orbital contains open space, LUMO tends to take an electron (Figure 3).

Compound with the smallest energy gap would be the reactive one among all. While the compound with the best kinetic stability is the compound showing great energy gap among all. The compound with the highest HOMO energy is the compound that would be the best electron donor. Whether the compound showing lowest LUMO value is the compound that would be the best electron acceptor. In current scenario, compound **6g** would be the highly reactive with energy gap of 0.142 eV. The most kinetically stable compound is **6i** with HOMO/LUMO energy gap of 0.147 eV. Compound **6e** would be the best electron donor with the highest HOMO energy of −0.121. Compound **6i** would be the best electron accepter with the lowest LUMO energy of 0.016. Energetic parameters are shown in Table 2.

**Table 2.** Energetic parameters that predict reactivity of compounds.

Compound	E_HOMO_ (eV)	E_LUMO_ (eV)	ΔE_gap_ (eV)	Hardness (η)	Softness (S)
**6a**	−0.12215	0.02109	0.1432	0.072	6.98
**6b**	−0.12197	0.02102	0.1430	0.071	6.99
**6c**	−0.12197	0.02109	0.1431	0.072	6.99
**6d**	−0.12188	0.02107	0.1430	0.071	7.00
**6e**	−0.12190	0.02118	0.1431	0.072	6.99
**6f**	−0.12189	0.02108	0.1430	0.071	6.99
**6g**	−0.12368	0.01890	0.1426	0.071	7.01
**6h**	−0.12235	0.01975	0.1421	0.071	7.04
**6i**	−0.13031	0.01668	0.1470	0.073	6.80
**6j**	−0.13625	0.00330	0.1396	0.070	7.17

### Alkaline phosphatase (ALPs) inhibition assay

The ten synthesised derivatives were subjected to evaluation for alkaline phosphatase inhibition. KH_2_PO_4_ was the reference substance used for screening, and Table 3 summarises the findings of evaluation as IC_50_. Interestingly, most of the synthesised compounds demonstrated promising inhibitory potential against alkaline ALPs. Moreover, most of the compounds showed better inhibition activity comparative to KH_2_PO_4._ Compound **6g** was compared to other analogues in the series, and was found as series’ significant alkaline phosphatase inhibitor.

**Table 3.** IC_50_ values of compounds **6**(**a–j**) values of alkaline phosphatase inhibitory activity.

Compound	Alkaline phosphatase	Compound	Alkaline phosphatase
	IC_50_ ± SEM (µM)		IC_50_ ± SEM (µM)
**6a**	7.247 ± 0.435	**6f**	8.681 ± 0.908
**6b**	3.154 ± 0.251	**6g**	0.337 ± 0.015
**6c**	4.239 ± 0.241	**6h**	6.304 ± 0.634
**6d**	0.886 ± 0.074	**6i**	0.957 ± 0.071
**6e**	0.754 ± 0.055	**6j**	0.509 ± 0.036
KH_2_PO_4_	5.245 ± 0.477		

Values are presented as Mean ± SEM (Standard error of mean).

### Structure activity relationship (SAR)

In Scheme 1, total 10 derivatives of the (*E*)-*N*-(4-(4-bromophenyl)-3-(quinolin-3-yl)thiazol-2(3*H*)-ylidene)alkyl/aryl amides **6(a–j)** were synthesised bearing various alkyl or aryl group substitution. The nature of functionalities substituted around iminothiazoline moiety influenced the alkaline phosphatase inhibition activity. Quinolinyl iminothiazoline moiety substituted with aromatic substituents (**6g–6j**) exhibit better inhibition activity comparative to aliphatic analogues (**6a–6f**). Among the analogues **6g–6j**, the compound **6g** having unsubstituted benzene ring proved as most potential inhibitor which might be due to less steric crowd. The compound **6g** (IC_50_ = 0.337 ± 0.015 µM) containing benzoyl moiety around iminothiazoline component exhibited greater inhibition potential among all other equivalents. The analogue **6j** (IC_50_ = 0.509 ± 0.036 µM) was retrieved as second best inhibitor due to the presence substituted aryl ring instead of long alkyl chain. However, the substitution on the aryl ring slightly reduced the inhibitory effect due to enhanced steric hindrance. The inhibitory activities of both phenyl bearing compounds against alkaline phosphatase was stronger than standard KH_2_PO_4_ (5.245 ± 0.477 µM). While in the aliphatic analogue (**6a–6f**), long alkyl chains, which are sterically hindered substituents, reduced the potential inhibitory effect. The compound **6f** (IC_50_ = 8.681 ± 0.908 µM) showed relatively low inhibitory efficacy, perhaps as a result of its lengthy alkyl chain. However, compounds **6d** and **6e** possessing hexyl and heptyl substituents, respectively, exhibited a comparatively better inhibition profile than standard compounds. The inhibitory activities of compound **6d** and **6e** were 0.886 ± 0.074 and 0.754 ± 0.055 µM respectively. Table 3 is representing inhibition profile of all synthesised derivatives.

### Kinetic mechanism for ALPs

**Figure 4.**
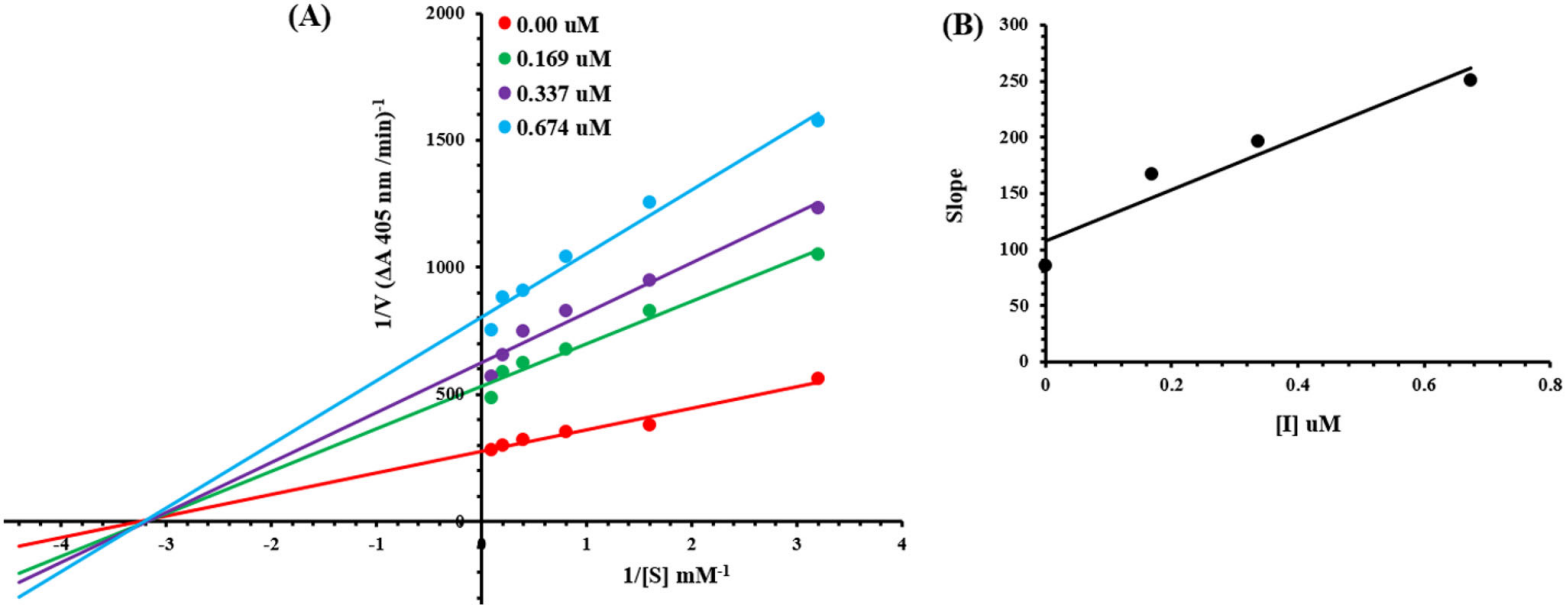
Alkaline phosphatase inhibition by compounds 6g as shown by Lineweaver-Burk plots (a). The plot of the slope vs inhibitor concentrations to get the inhibition constant is shown in the insets (b). Using the linear least squares fit, the lines were drawn.

The current most effective compound, **6g**, has been investigated for its mechanism of alkaline phosphatase inhibition. According to the EI and ESI constants, respectively, it was determined if the compounds had the ability to inhibit the free enzyme and the enzyme-substrate complex. Figure 4 illustrates a succession of straight lines obtained from the Lineweaver–Burk plot of the enzyme’s kinetic studies against the substrate para nitrophenyl phosphate disodium salt at various inhibitor doses (A). Compounds **6g**’s outcomes demonstrated that they intersected in the 2nd quadrant. The results exhibited that whereas K_m_ remained constant, V_max_ fell in response to new, increasing inhibitor doses. This behaviour suggests that compounds **6g** produce an enzyme inhibitor complex by non-competitively inhibiting alkaline phosphatase. The secondary plot of slope vs inhibitor concentration shown in Figure 4 revealed the enzyme–inhibitor dissociation constant (*Ki*) (B). Table 4 displays the kinetic results (Kinetic parameter table).

**Table 4.** Kinetic parameters of the alkaline phosphatase for para nitrophenyl phosphate disodium salt activity in the presence of different concentrations of **6g**.

Concentration (µM)	V_max_ (ΔA /Min)	K_m_ (mM)	Inhibition type	*K*i (µM)
0.00	0.00354	0.31	Non-Competitive	0.47
0.169	0.00204	0.31		
0.337	0.00175	0.31		
0.674	0.00133	0.31		

*K*i = EI dissociation constant; V_max_ = the reaction velocity; K_m_ = Michaelis-Menten constant.

### Molecular docking

The Molecular Operating Environment (MOE) program was used to determine the most likely molecular interactions between ligands and targeted protein. The synthesised derivatives demonstrated potential *in-vitro* activities were selected for evaluation of binding mode inside active pocket of protein. The amino acid residues engaged in the molecular interactions were as follows; His331, Asp327, Asn263, Lys328, Asp153, Lys167, Tyr169, Arg166, Gly118, Asn117, Asp101, Glu411, His412, Val99, Arg24, Arg62, Arg10, Asp76, Arg418, Phe71, Thr81, Leu25, Ser409, Gln410, Ser409, Lys328, Tyr169, and Arg166. The docking score of compound **6d**, **6e**, **6g**, **6i** and **6j** were tabulated in Table 5.

**Table 5.** Binding energies of the potent inhibitors of alkaline phosphatase.

Compound	Binding energies (kJ/mol)	Hydrogen bonding residues	Bond length (angstroms)
**6d**	−26.75	Lys328, Arg166	3.27, 3.04
**6e**	−25.94	His412	3.09
**6g**	−28.86	Ser409	2.93
**6i**	−25.10	–	–
**6j**	−28.45	Ser409, Ser409, Arg166	3.0, 3.0, 3.03

The docked conformation analysis for compound **6d** exhibited strong molecular interactions. Conclusively, two hydrogen bonds were observed with the targeted protein. The first hydrogen bond was present between the electronegative oxygen atom and Arg166 with a bond length of 3.04 angstroms. A second hydrogen bond was observed between N3 of compound **6** and Asn263, with a bond length of 3.27 angstroms. In addition, hydrophobic interactions were engaging His 331, Asp327, Asn263, Lys167, Tyr169, and Asp153. These interactions were collectively stabilising the protein–ligand complex with a docking score of −26.75 kJ/mol.

The binding orientation of compound 6e exhibited potential molecular interactions with the targeted protein. It was observed that compound **6e** was involved in single hydrogen bonding between electronegative oxygen and His412 residue. The bond length of hydrogen bonding was 3.09 angstroms, indicating its good strength. The docking score of conformation was −25.96 kJ/mol, which is slightly lower than compound **6d**. It was expected because compound **6D** formed strong hydrogen bonds. In addition, the following amino acid residues were involved in hydrophobic interactions with compound **6e**: Arg166, Gly118, Asn117, Asp101, Glu411, His412, and Val99.

The analysis of the binding orientation of compound **6g** demonstrated significant molecular interactions and exhibited the highest docking score. Interestingly, compound **6G** demonstrated potential *in vitro* activity against alkaline phosphatase, which is further supported by in silico findings. Critical analysis revealed that compound **6G** involved important amino residues at the active site with strong molecular interactions. A single hydrogen bond was observed between electronegative oxygen and Ser409 with a strong bond length of 2.93 angstroms. Furthermore, hydrophobic interactions involved His331, Gln410, Glu411, Asp101, and His412 amino acid residues with strong strength. The docking score of the protein-**6g** conformation was −28.86 kJ/mol.

**Figure 5.**
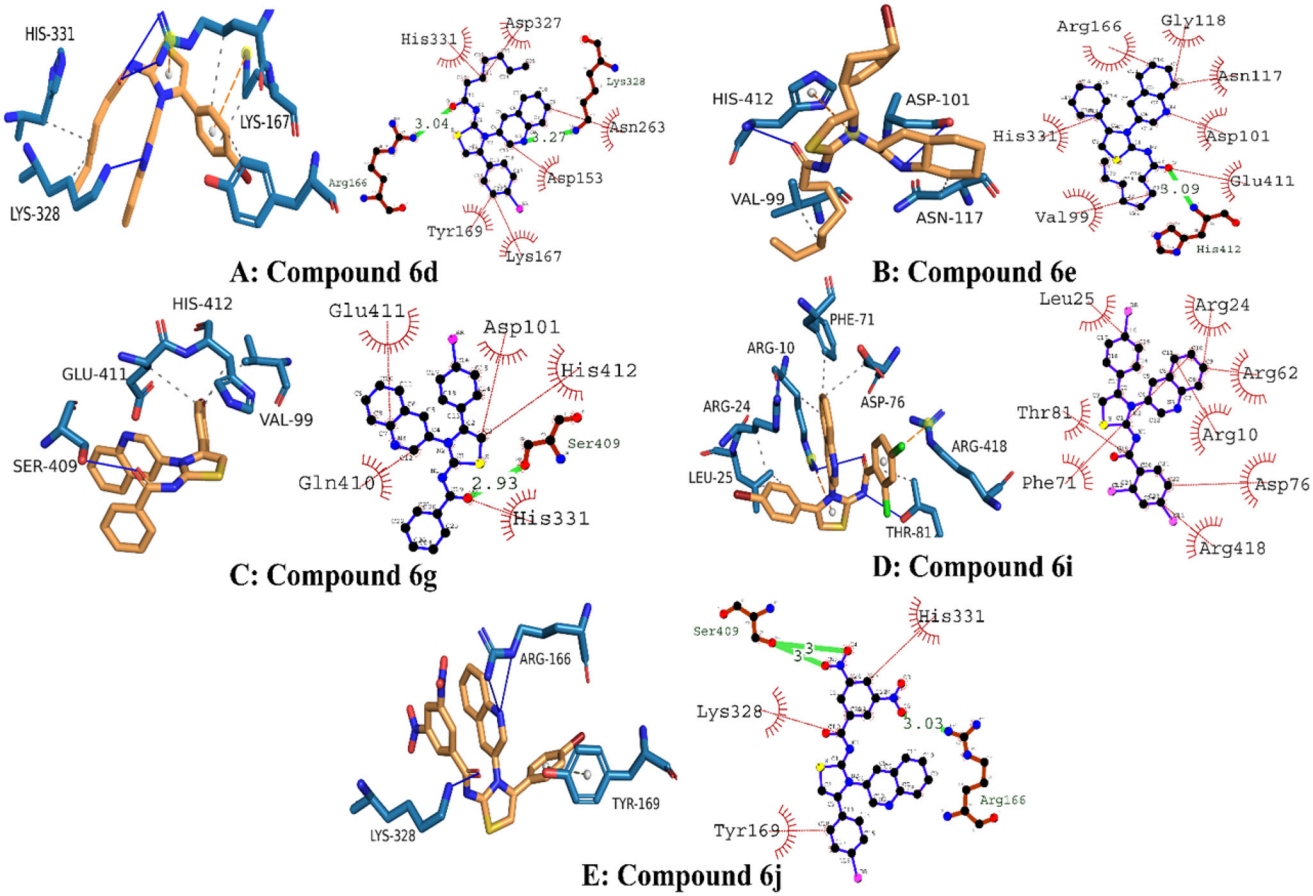
Illustrating the 3D and 2D binding interactions of potent compounds (**6d**, **6e**, **6g**, **6i and 6j**).

In terms of molecular interactions, compounds **6i** and **6j** exhibited strong chemical bonding with targeted proteins. However, the chemical bonding strength of compound **6j** was stronger than compound **6i**. Briefly, it was observed that compound **6i** was engaging Arg24, Arg62, Arg10, Asp76, Arg418; Phe71; Thr81; and Leu25 amino acid residues in important molecular interactions, and the docking score of conformation was observed to be −25.10 kJ/mol. Whereas, compound **6j** demonstrated equipotent activity with compound **6g** with a docking score of −28.45 kJ/mol. It was observed that a total of three hydrogen bonds were produced by compound **6j**. Electronegative oxygen atoms O_4_ and O_5_ were engaging Ser409 in hydrogen bonding with bond lengths of 3 and 3 angstroms, respectively. Another hydrogen bond was observed between O_2_ of compound **6j** and Arg166 of the targeted protein. These hydrophilic interactions stabilised the protein–ligand complex. In addition, His331, Lys328, and Tyr169 were engaged in hydrophobic interactions, including alkyl, pi-alkyl, and aromatic interactions, respectively. The presumed 3D and 2D interactions of top-ranked compounds **6d**, **6e**, **6g**, **6i**, and **6j** are illustrated in Figure 5.

### Molecular Dynamics simulation studies

**Figure 6.**
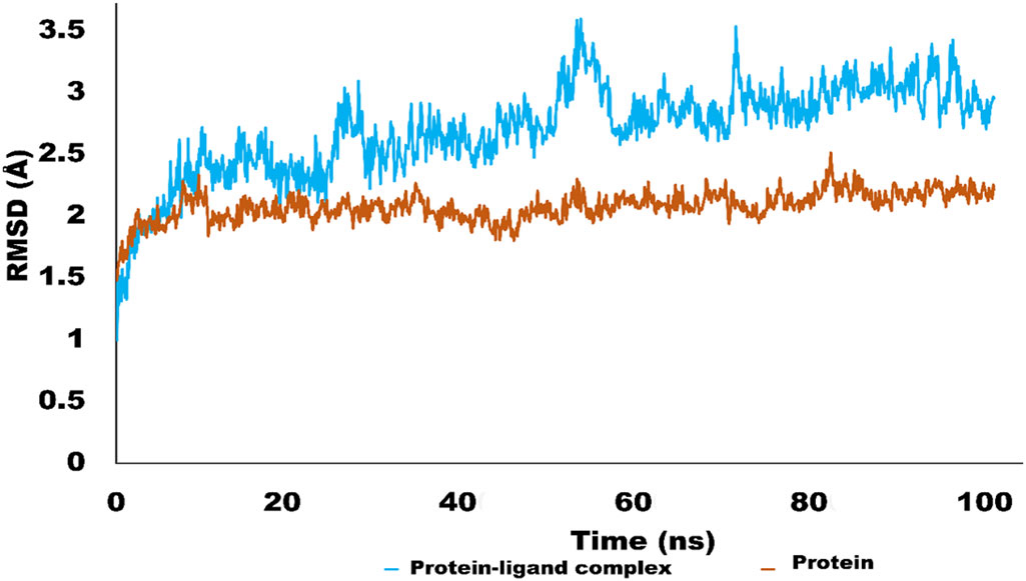
RMSD trajectory analysis for protein (brown coloured trajectory), protein ligand complex (blue coloured trajectory).

The Molecular dynamics simulations are used to estimate the thermodynamic parameters of living systems under physiological conditions. The best docked conformation was subjected to MD simulations studies using Desmond program. The simulation studies for 100 ns provided significant insight into stability of protein ligand complex. MD simulation trajectories were used to derive various analytic matrices including RMSD, RMSF, contact profile and ligand interaction profile. The RMSD plot for protein(C alpha) and protein–ligand complex is represented in Figure 6. The RMSD pattern for C alpha atoms of protein exhibited significant stability. It was revealed that initially RMSD fluctuate around 2 Å which rose up to 2.4 Å. The amino acid residues Thr17, Gly14, Asn15, Gln13, Gly27 and Thr1 exhibited variations up to 2.4 Å. However, the average RMSD for protein C alpha atom was 2.01 Å which is perfectly in acceptable limit. In terms of complex RMSD, it was slightly higher than c-alpha atoms of protein. Initially complex fluctuated around 2–2.5 Å which jumped to 3.5 Å after 50 ns of simulation. Interestingly, after rising to 3.5 Å, RMSD again dropped to 2.5 Å and get stable and equilibrated throughout the trajectory. The higher fluctuations of complex were due to structural changes of ligand inside active pocket. After 50 ns, ligand established new and stronger interactions which stabilised the trajectory during the simulated time. The average RMSD of the complex was found to be 2.69 Å which is acceptable^69^. The Figure 6 is illustrating the RMSD pattern for protein and protein–ligand complex.

**Figure 7.**
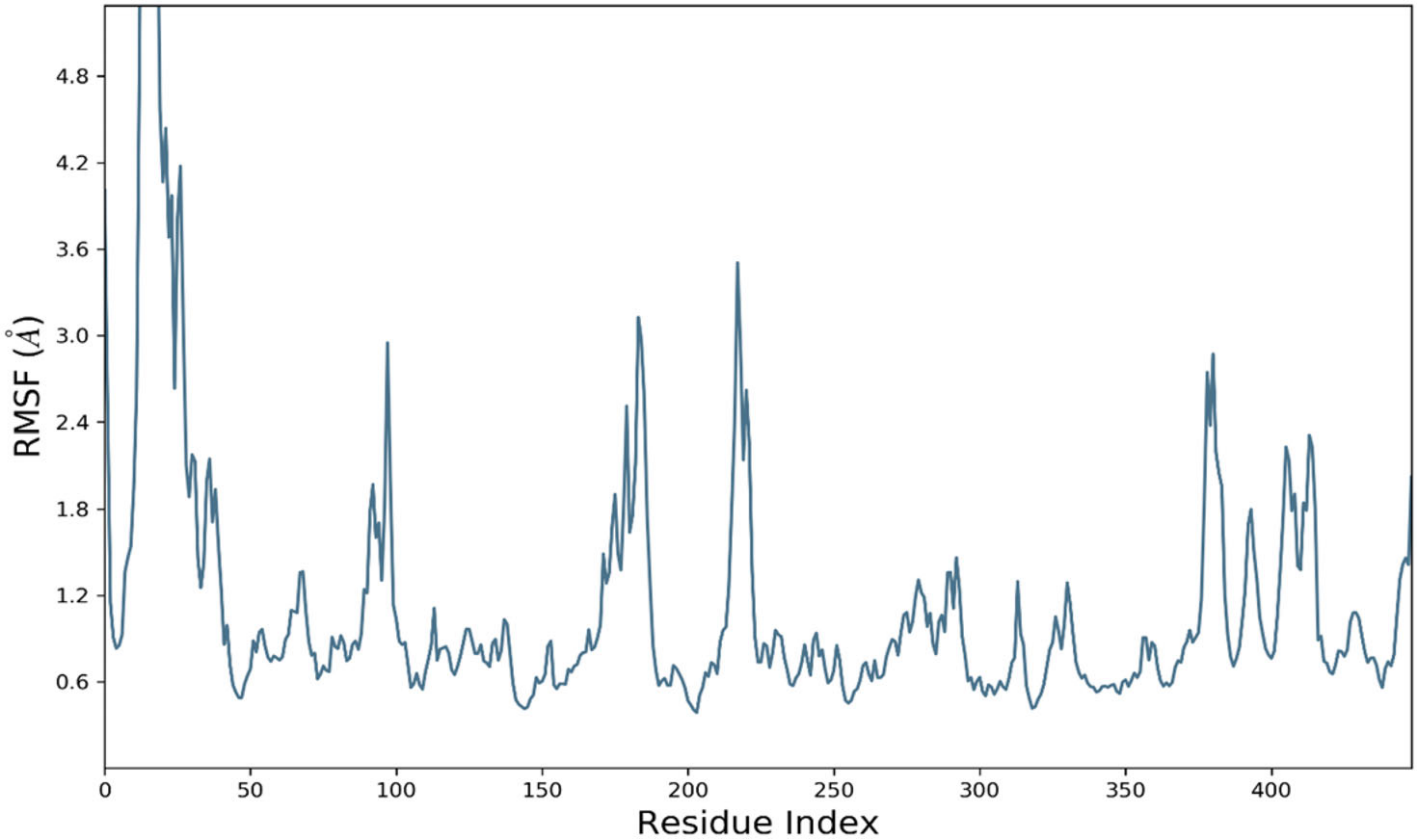
Evolution of RMSF for amino acid residues of targeted protein.

The analysis of root mean square fluctuations (RMSF) is crucial analytical metric in characterising the local structural changes in the protein. In current study, residue wise RMSF was generated for each residue. RMSF exhibited optimal fluctuations for majority of residues but residues Thr1, Asn15, Ile16, THr17, Ala18, Pro19, Gly20, Gly21, Ala22, and Arg23 exhibited substantial structural variations up to 8 Å. These residues were belongs to C and N terminal which are comparatively less compact than backbone and alpha strands residues. The average RMSF value for whole protein was calculated as 1.14 Å which is quite acceptable. The RMSF evolution is presented in Figure 7.

**Figure 8.**
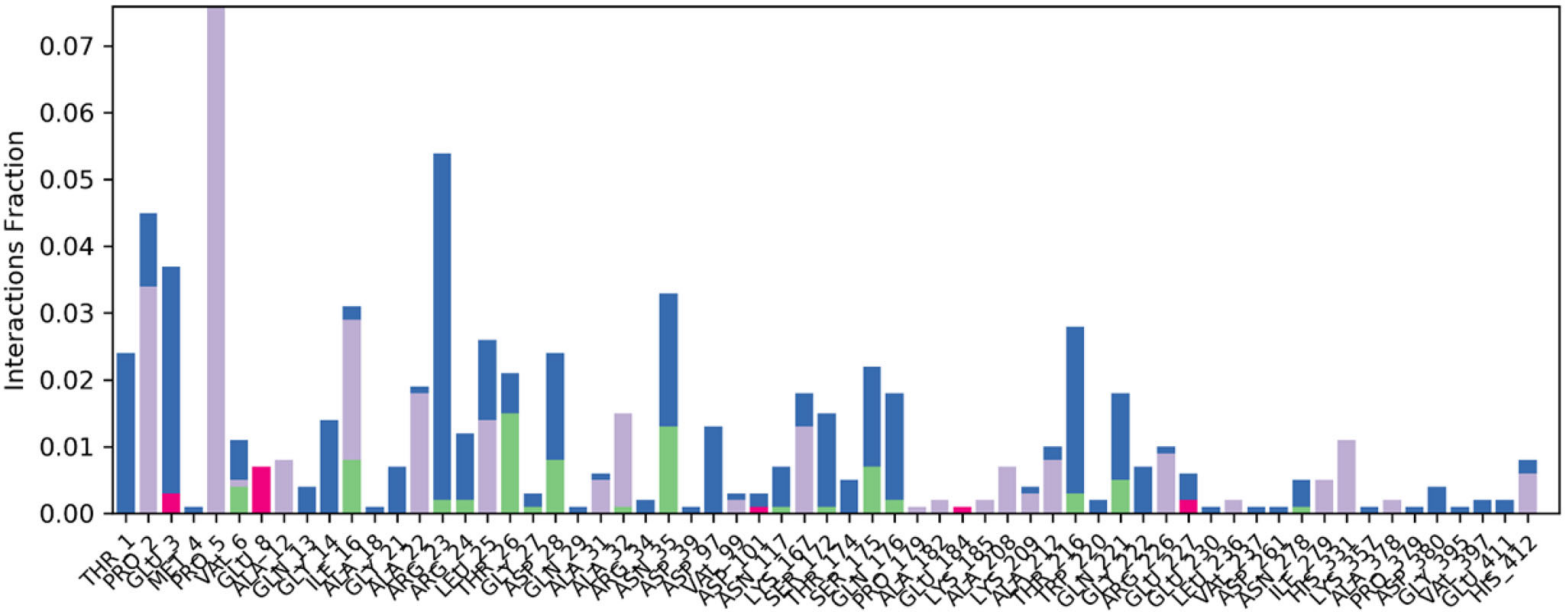
Contact profile for protein-ligand complex. Green coloured peaks are representing hydrogen bonding while purple peaks are representing hydrophobic interactions. Blue coloured histograms are water bridges.

The contact linkages play important role in establishing the significant contacts between ligand and protein. Type and interaction time determine the stability of complex. In present complex, hydrogen bonding, water bridges and hydrophobic interactions were observed between protein and ligand. Important hydrogen bonding was observed between ligand and Asn35 and Thr26 of targeted protein. Hydrogen bonding was established for more than 15% of simulation time. In terms of hydrophobic interactions, Pro1 and Pro5 established significant hydrophobic contacts for 30% and 80% of simulation time respectively. These interactions were contributing substantially in stabilising the protein ligand complex. Figure 8 is representing the contact profile for simulated complex.

**Figure 9.**
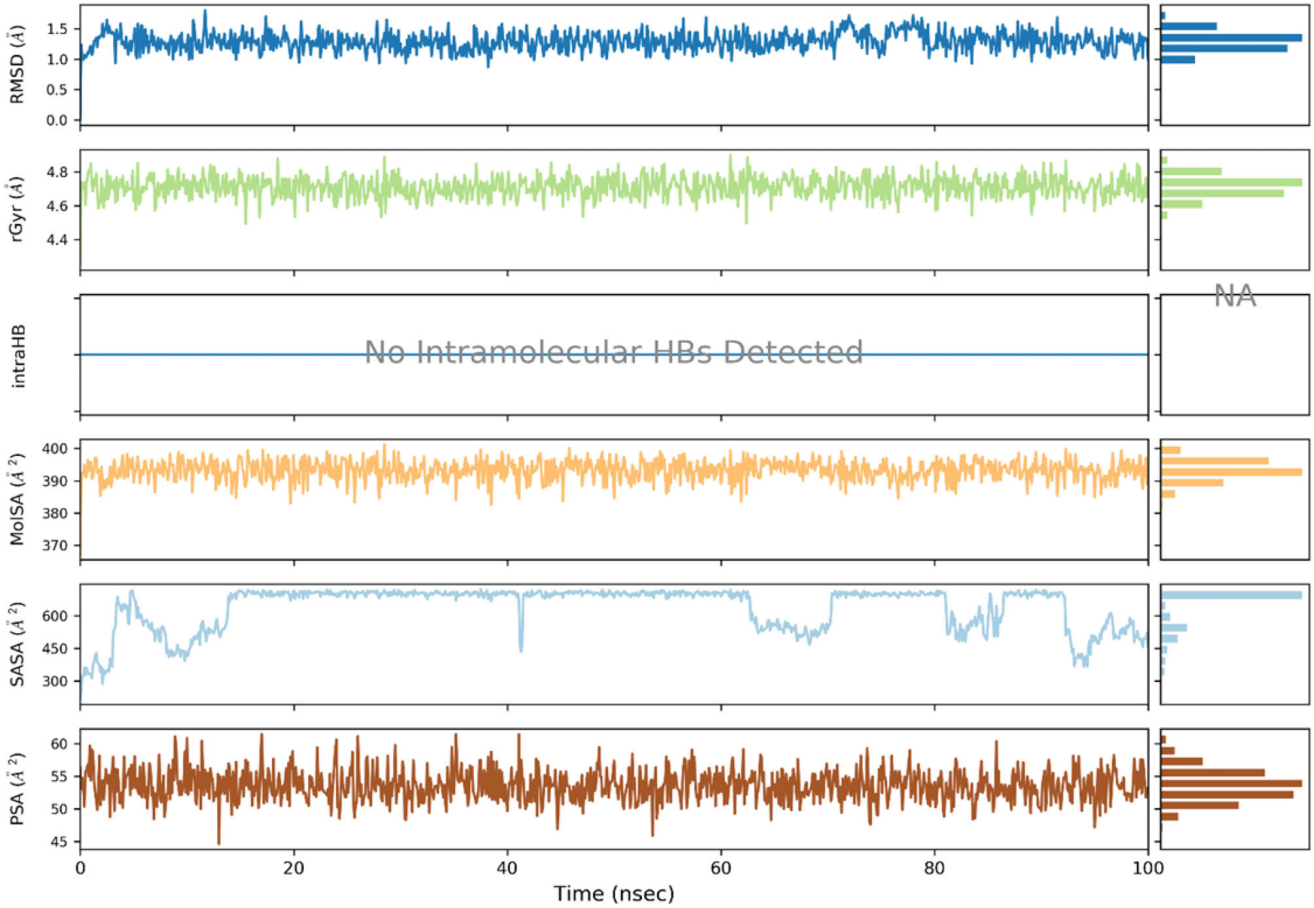
Ligand properties analysed using various analytical metrics.

Ligand properties were also evaluated to determine its stability and variation profile. Atomic fluctuations, solvent accessibility and compactness of ligand remained in the acceptable limit. The ligand properties are illustrated in the Figure 9.

**Figure 10.**
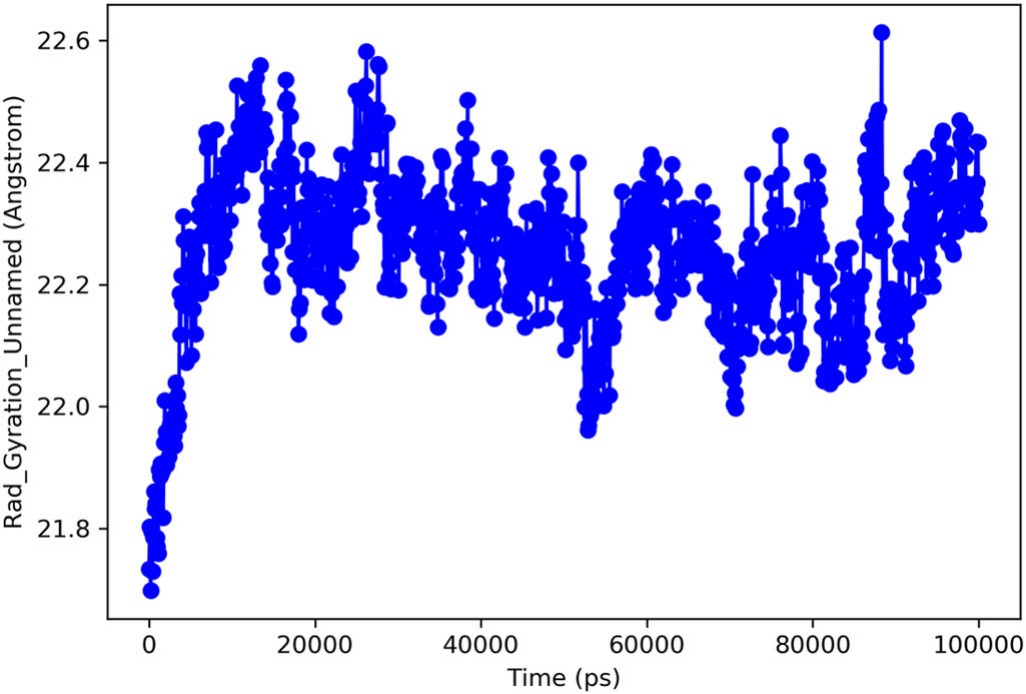
Radius of gyration.

Radius of gyration (Rg) is important analytic metrics for determination of centre of mass and compactness of protein. A low value for Rg depicts the high compactness and low structural variations of the protein. Whereas, high Rg is predictive of poor stability and more structural changes of a particular protein. In current study, protein exhibited optimal structural changes with Rg ranges for 21.8–22.6 Å. These findings predict that mass of protein was equally distributed around single point and remained well compact through simulated trajectory. Figure 10 is illustrating the Rg for targeted protein.

## Conclusions

The quinolinyl based iminothiazoline analogues **6(a–j)** containing alkyl and aryl groups were prepared and characterised by FT-IR, ^1^H NMR, ^13^C NMR and HPLC-MS. All the analogues were evaluated for alkaline phosphatase inhibition potential. The compound (*E*)-*N*-(4-(4-bromophenyl)-3-(quinolin-3-yl)thiazol-2(3H)-ylidene)benzamide **6g** exhibited the maximum alkaline phosphatase inhibitory effect (IC_50_ = 0.337 ± 0.015 µM comparative to other synthesised derivatives and reference compound KH_2_PO_4_ (IC_50_ = 5.245 ± 0.477 µM). DFT studies were performed and compounds **6j**, **6g** and **6h** were found to be the reactive ones among all compounds. Kinetic analysis exhibited that the analogue (**6g**) was non-competitive inhibitor of alkaline phosphatase with Ki value of 0.47 µM. The molecular docking displayed that the compounds **6d**, **6e**, **6g** and **6i** possesses efficient binding affinity but we considered **6g** because the result of docking is proved confirmatory for kinetic analysis as well that our **6g** compound is a good inhibitor of the targeted protein alkaline phosphatase. Comprehensive MD investigations were carried out in an effort to further evaluate the validity of docking data, further validating the inhibitory capability of **6g**. The quinolinyl and aryl or alkyl moiety in iminothiazolines play significant role in alkaline phosphatase inhibition potential. The derivative **6g** may be considered to design more potent drug for the alkaline phosphatase inhibition.

## Supplementary Material

Supplemental Material

## Data Availability

All research data will be made available on request.

## References

[1] Straus DS, Glass CK. Anti-inflammatory actions of PPAR ligands: new insights on cellular and molecular mechanisms. Trends Immunol. 2007;28(12):551–558. 17981503 10.1016/j.it.2007.09.003

[2] Chang TC, Wang JK, Hung MW, Chiao CH, Tsai LC, Chang GG. Regulation of the expression of alkaline phosphatase in a human breast-cancer cell line. Biochem J. 1994;303(1):199–205. 7945240 10.1042/bj3030199 PMC1137576

[3] al‐Rashida M, Iqbal J. Therapeutic potentials of ecto‐nucleoside triphosphate diphosphohydrolase, ecto‐nucleotide pyrophosphatase/phosphodiesterase, ecto‐5′‐nucleotidase, and alkaline phosphatase inhibitors. Med Res Rev. 2014;34(4):703–743. 24115166 10.1002/med.21302

[4] Ali AT, Penny CB, Paiker JE, Psaras G, Ikram F, Crowther NJ. The effect of alkaline phosphatase inhibitors on intracellular lipid accumulation in preadipocytes isolated from human mammary tissue. Ann Clin Biochem. 2006;43(Pt 3):207–213. 16704756 10.1258/000456306776865179

[5] Llinas P, Stura EA, Ménez A, Kiss Z, Stigbrand T, Millán JL, Le Du MH. Structural studies of human placental alkaline phosphatase in complex with functional ligands. J Mol Biol. 2005;350(3):441–451. 15946677 10.1016/j.jmb.2005.04.068

[6] Sundström B, Johansson B, Hietala SO, Stigbrand T. Radio-immunolocalization in nude mice using anticytokeratin monoclonal antibodies. Tumour Biol. 1990;11(3):158–166. 1693011 10.1159/000217650

[7] Orsaria M, Londero AP, Marzinotto S, Di Loreto C, Marchesoni D, Mariuzzi L. Placental type alkaline phosphatase tissue expression in ovarian serous carcinoma. Cancer Biomark. 2016;17(4):479–486. 27802199 10.3233/CBM-160665 PMC13020508

[8] Hanschkow M, Boulet N, Kempf E, Bouloumié A, Kiess W, Stein R, Körner A, Landgraf K. Expression of the adipocyte progenitor markers MSCA1 and CD36 is associated with adipose tissue function in children. J Clin Endocrinol Metab. 2022;107(2):e836–e851. 34448000 10.1210/clinem/dgab630 PMC8764220

[9] Tuin A, Poelstra K, de Jager-Krikken A, Bok L, Raaben W, Velders MP, Dijkstra G. Role of alkaline phosphatase in colitis in man and rats. Gut. 2009;58(3):379–387. 18852260 10.1136/gut.2007.128868

[10] Su F, Brands R, Wang Z, Verdant C, Bruhn A, Cai Y, Raaben W, Wulferink M, Vincent JL. Beneficial effects of alkaline phosphatase in septic shock. Crit Care Med. 2006;34(8):2182–2187. 16775571 10.1097/01.CCM.0000229887.70579.29

[11] Alam SN, Yammine H, Moaven O, Ahmed R, Moss AK, Biswas B, Muhammad N, Biswas R, Raychowdhury A, Kaliannan K, et al. Intestinal alkaline phosphatase prevents antibiotic-induced susceptibility to enteric pathogens. Ann Surg. 2014;259(4):715–722. 23598380 10.1097/SLA.0b013e31828fae14 PMC3855644

[12] Anderson HC. The role of matrix vesicles in physiological and pathological calcification. Curr Opin Orthop. 2007;18(5):428–433.

[13] van der Heijde D, Kivitz A, Schiff MH, Sieper J, Dijkmans BAC, Braun J, Dougados M, Reveille JD, Wong RL, Kupper H, ATLAS Study Group, et al. Efficacy and safety of adalimumab in patients with ankylosing spondylitis: results of a multicenter, randomized, double-blind, placebo-controlled trial. Arthritis Rheum. 2006;54(7):2136–2146. 16802350 10.1002/art.21913

[14] Brandt J, Khariouzov A, Listing J, Haibel H, Sörensen H, Grassnickel L, Rudwaleit M, Sieper J, Braun J. Six‐month results of a double‐blind, placebo‐controlled trial of etanercept treatment in patients with active ankylosing spondylitis. Arthritis Rheum. 2003;48(6):1667–1675. 12794835 10.1002/art.11017

[15] van der Heijde D, Dijkmans B, Geusens P, Sieper J, DeWoody K, Williamson P, Braun J, Ankylosing Spondylitis Study for the Evaluation of Recombinant Infliximab Therapy Study Group. Efficacy and safety of infliximab in patients with ankylosing spondylitis: results of a randomized, placebo‐controlled trial (ASSERT). Arthritis Rheum. 2005;52(2):582–591. 15692973 10.1002/art.20852

[16] Antoni C, Krueger GG, de Vlam K, Birbara C, Beutler A, Guzzo C, Zhou B, Dooley LT, Kavanaugh A, IMPACT 2 Trial Investigators. Infliximab improves signs and symptoms of psoriatic arthritis: results of the IMPACT 2 trial. Ann Rheum Dis. 2005;64(8):1150–1157. 15677701 10.1136/ard.2004.032268 PMC1755609

[17] Li L, Chang L, Pellet-Rostaing S, Liger F, Lemaire M, Buchet R, Wu Y. Synthesis and evaluation of benzo [b] thiophene derivatives as inhibitors of alkaline phosphatases. Bioorg Med Chem. 2009;17(20):7290–7300. 19781951 10.1016/j.bmc.2009.08.048

[18] Narisawa S, Harmey D, Yadav MC, O'Neill WC, Hoylaerts MF, Millán JL. Novel inhibitors of alkaline phosphatase suppress vascular smooth muscle cell calcification. J Bone Miner Res. 2007;22(11):1700–1710. 17638573 10.1359/jbmr.070714

[19] Maslat AO, Abussaud M, Tashtoush H, Al-Talib M. Synthesis, antibacterial, antifungal and genotoxic activity of bis-1, 3, 4-oxadiazole derivatives. Pol J Pharmacol. 2002;54(1):55–59. 12020044

[20] Barsu N, Sen M, Premkumar JR, Sundararaju B. Cobalt (iii) catalyzed C–8 selective C–H and C–O coupling of quinoline N-oxide with internal alkynes via C–H activation and oxygen atom transfer. Chem Commun (Camb)). 2016;52(7):1338–1341. 26695530 10.1039/c5cc08736h

[21] Baird JK, Rieckmann KH. Can primaquine therapy for vivax malaria be improved? Trends Parasitol. 2003;19(3):115–120. 12643993 10.1016/s1471-4922(03)00005-9

[22] Hargrave KD, Hess FK, Oliver JT. N-(4-Substituted-thiazolyl) oxamic acid derivatives, new series of potent, orally active antiallergy agents. J Med Chem. 1983;26(8):1158–1163. 6876084 10.1021/jm00362a014

[23] Fisher RS, van Emde Boas W, Blume W, Elger C, Genton P, Lee P, Engel J. Epileptic seizures and epilepsy: definitions proposed by the International League Against Epilepsy (ILAE) and the International Bureau for Epilepsy (IBE). Epilepsia. 2005;46(4):470–472. 15816939 10.1111/j.0013-9580.2005.66104.x

[24] El-Ansary SL, Hassan GS, Abdel Rahman DE, Farag NA, Hamed MI, Baset MA. Design, synthesis and biological evaluation of some new succinimide, 2-iminothiazoline and oxazine derivatives based benzopyrone as anticonvulsant agents. Int J Pharm Pharm Sci. 2016;8(4):222–228.

[25] Larik FA, Saeed A, Faisal M, Hamdani S, Jabeen F, Channar PA, Mumtaz A, Khan I, Kazi MA, Abbas Q, et al. Synthesis, inhibition studies against AChE and BChE, drug-like profiling, kinetic analysis and molecular docking studies of N-(4-phenyl-3-aroyl-2 (3H)-ylidene) substituted acetamides. J Mol Struct. 2020;1203:127459.

[26] Sharma PK, Sawhney SN, Gupta A, Singh GB, Bani S. Synthesis and antiinflammatory activity of some 3-(2-thiazolyl)-1, 2-benzisothiazoles. Indian J Chem B. 1998;37:376–381.

[27] Saeed A, Al-Masoudi NA, Pannecouque C. In-vitro anti-HIV activity of new thiazol-2-ylidene substituted benzamide analogues. Der Pharma Chem. 2012;4(1):106–115.

[28] Sondhi SM, Singh N, Lahoti AM, Bajaj K, Kumar A, Lozach O, Meijer L. Synthesis of acridinyl-thiazolino derivatives and their evaluation for anti-inflammatory, analgesic and kinase inhibition activities. Bioorg Med Chem. 2005;13(13):4291–4299. 15927836 10.1016/j.bmc.2005.04.017

[29] Tsuji K, Ishikawa H. Synthesis and anti-pseudomonal activity of new 2-isocephems with a dihydroxypyridone moiety at C-7. Bioorg Med Chem Lett. 1994;4(13):1601–1606.

[30] Saeed S, Hussain R, Ali M. Synthesis and Antimicrobial Activity of N‐[(2 Z)‐3‐(4, 6‐Substitutedpyrimidin‐2‐yl)‐4‐phenyl‐1, 3‐thiazol‐2 (3 H)‐ylidene]‐3, 5‐dinitrobenzamide Analogues. J Heterocyclic Chem. 2013;50(2):237–243.

[31] Rokach J, Girard Y, Hamel P, Reader G, Rooney CS, Mandel LR, Cragoe EJ, Zacchei AG. Inhibitors of indoleethylamine N-methyltransferase. Derivatives of 3-methyl-2-thiazolidinimine. *In vitro*, *in vivo*, and metabolic studies. J Med Chem. 1980;23(7):773–780. 7401104 10.1021/jm00181a014

[32] Saeed A, Rafique H. Synthesis of new N-[3-(Benzo [d] thiazol-2-yl)-4-methylthiazol-2 (3H)-ylidene] substituted benzamides. Turk J Chem. 2013;37(6):909–916.

[33] Tomizawa M, Cowan A, Casida JE. Analgesic and toxic effects of neonicotinoid insecticides in mice. Toxicol Appl Pharmacol. 2001;177(1):77–83. 11708903 10.1006/taap.2001.9292

[34] Hosseinimehr SJ, Shafiee A, Mozdarani H, Akhlagpour S. Radioprotective effects of 2-iminothiazolidine derivatives against lethal doses of gamma radiation in mice. J Radiat Res. 2001;42(4):401–408. 11951663 10.1269/jrr.42.401

[35] Chowdhury R, Candela-Lena JI, Chan MC, Greenald DJ, Yeoh KK, Tian YM, McDonough MA, Tumber A, Rose NR, Conejo-Garcia A, et al. Selective small molecule probes for the hypoxia inducible factor (HIF) prolyl hydroxylases. ACS Chem Biol. 2013;8(7):1488–1496. 23683440 10.1021/cb400088q

[36] Manaka A, Ishii T, Takahashi K, Sato M. 2-Acylimino-3-alkyl-3H-thiazoline derivatives: one-pot, three-component condensation synthesis of novel β-turn mimics. Tetrahedron Lett. 2005;46(3):419–422.

[37] Kim DS, Jeong YM, Park IK, Hahn HG, Lee HK, Kwon SB, Jeong JH, Yang SJ, Sohn UD, Park KC, et al. A new 2-imino-1, 3-thiazoline derivative, KHG22394, inhibits melanin synthesis in mouse B16 melanoma cells. Biol Pharm Bull. 2007;30(1):180–183. 17202683 10.1248/bpb.30.180

[38] Pietrancosta N, Moumen A, Dono R, Lingor P, Planchamp V, Lamballe F, Bähr M, Kraus JL, Maina F. Imino-tetrahydro-benzothiazole derivatives as p53 inhibitors: discovery of a highly potent *in vivo* inhibitor and its action mechanism. J Med Chem. 2006;49(12):3645–3652. 16759106 10.1021/jm060318n

[39] Heravi MM, Moghimi S. An efficient synthesis of thiazol-2-imine derivatives via a one-pot, three-component reaction. Tetrahedron Lett. 2012;53(4):392–394.

[40] Assis DB, Aragão Neto HC, da Fonsêca DV, de Andrade HHN, Braga RM, Badr N, Maia MDS, Castro RD, Scotti L, Scotti MT, et al. Antinociceptive activity of chemical components of essential oils that involves docking studies: a review. Front Pharmacol. 2020;11:777. 32547391 10.3389/fphar.2020.00777 PMC7272657

[41] Chang L, Duy DL, Mébarek S, Popowycz F, Pellet-Rostaing S, Lemaire M, Buchet R. Synthesis and evaluation of thiophenyl derivatives as inhibitors of alkaline phosphatase. Bioorg Med Chem Lett. 2011;21(8):2297–2301. 21421310 10.1016/j.bmcl.2011.02.089

[42] Azarakhshi F, Khaleghian M, Farhadyar N. DFT study and NBO analysis of conformational properties of 2-substituted 2-Oxo-1, 3, 2-dioxaphosphorinanes and their dithia and diselena analogs. Lett Org Chem. 2015;12(7):516–522.

[43] Frisch M, Trucks GW, Schlegel HB, Scuseria GE, Robb MA, Cheeseman JR, Scalmani G, Barone V, Mennucci B, Petersson GA, et al. Gaussian 09, Revision B. 01. Wallingford (CT): Gaussian, Inc; 2009.

[44] Al-Rashida M, Iqbal J. Inhibition of alkaline phosphatase: an emerging new drug target. Mini Rev Med Chem. 2015;15(1):41–51. 25694083 10.2174/1389557515666150219113205

[45] Channar SA, Channar PA, Saeed A, Alsfouk AA, Ejaz SA, Ujan R, Noor R, Bilal MS, Abbas Q, Hussain Z, et al. Exploring thiazole-linked thioureas using alkaline phosphatase assay, biochemical evaluation, computational analysis and structure–activity relationship (SAR) studies. Med Chem Res. 2022;31(10):1792–1802.

[46] Bauernschmitt R, Häser M, Treutler O, Ahlrichs R. Calculation of excitation energies within time-dependent density functional theory using auxiliary basis set expansions. Chem Phys Lett. 1997;264(6):573–578.

[47] Calais JL. Orthonormalization and symmetry adaptation of crystal orbitals. Int J Quantum Chem. 2009;28(S19):655–667.

[48] Weigend F, Ahlrichs R. Balanced basis sets of split valence, triple zeta valence and quadruple zeta valence quality for H to Rn: design and assessment of accuracy. Phys Chem Chem Phys. 2005;7(18):3297–3305. 16240044 10.1039/b508541a

[49] Hossen J, Ali MA, Reza S. Theoretical investigations on the antioxidant potential of a non-phenolic compound thymoquinone: a DFT approach. J Mol Model. 2021;27(6):1–11. 10.1007/s00894-021-04795-0 34014420

[50] Parr RG, Yang W. Density functional theory of atoms and molecules. New York (NY): Oxford University Press; 1989: p. 1989.

[51] Schäfer A, Huber C, Ahlrichs R. Fully optimized contracted Gaussian basis sets of triple zeta valence quality for atoms Li to Kr. J Chem Phys. 1994;100(8):5829–5835.

[52] Bartolotti LJ, Flurchick K. An introduction to density functional theory. Rev Comput Chem. 1996;7:187–260.

[53] Thanikaivelan P, Subramanian V, Raghava Rao J, Unni Nair B. Application of quantum chemical descriptor in quantitative structure activity and structure property relationship. Chem Phys Lett. 2000;323(1–2):59–70.

[54] Dennington R, Keith T, Millam J, Eppinnett K, Hovell WL, Gilliland R. GaussView v.5.0.9 visualizer and builder. Wallingford (CT): Gaussian Inc; 2009.

[55] Iqbal Z, Ashraf Z, Hassan M, Abbas Q, Jabeen E. Substituted phenyl [(5-benzyl-1, 3, 4-oxadiazol-2-yl) sulfanyl] acetates/acetamides as alkaline phosphatase inhibitors: synthesis, computational studies, enzyme inhibitory kinetics and DNA binding studies. Bioorg Chem. 2019;90:103108. 31284102 10.1016/j.bioorg.2019.103108

[56] Saeed A, Saddique G, Ali Channar P, Ali Larik F, Abbas Q, Hassan M, Raza H, Fattah TA, Seo SY. Synthesis of sulfadiazinyl acyl/aryl thiourea derivatives as calf intestinal alkaline phosphatase inhibitors, pharmacokinetic properties, lead optimization, Lineweaver-Burk plot evaluation and binding analysis. Bioorg Med Chem. 2018;26(12):3707–3715. 29884581 10.1016/j.bmc.2018.06.002

[57] Abbasi MA, Nazir M, Ur-Rehman A, Siddiqui SZ, Hassan M, Raza H, Shah SAA, Shahid M, Seo SY. Bi‐heterocyclic benzamides as alkaline phosphatase inhibitors: mechanistic comprehensions through kinetics and computational approaches. Arch Pharm. 2019;352(3):1800278. 10.1002/ardp.201800278 30624805

[58] Molecular Operating Environment (MOE). Montreal (QC): Chem Comput Group Inc; 2016.

[59] Heinzerling L, Klein R, Rarey M. Fast force field‐based optimization of protein–ligand complexes with graphics processor. J Comput Chem. 2012;33(32):2554–2565. 22911510 10.1002/jcc.23094

[60] Al‐Hazmi GAA, Abou‐Melha KS, El‐Metwaly NM, Althagafi I, Shaaban F, Zaky R. Green synthesis approach for Fe (III), Cu (II), Zn (II) and Ni (II)‐Schiff base complexes, spectral, conformational, MOE‐docking and biological studies. Appl Organomet Chem. 2020;34(3):e5403.

[61] Yuan S, Chan HS, Hu Z. Using PyMOL as a platform for computational drug design. Wiley Interdiscip Rev Comput Mol Sci. 2017;7(2):e1298.

[62] Laskowski RA, Swindells MB. LigPlot+: multiple ligand–protein interaction diagrams for drug discovery. J Chem Inf Model. 2011;51(10):2778–2786. 21919503 10.1021/ci200227u

[63] Zhang Y, Zhang TJ, Tu S, Zhang ZH, Meng FH. Identification of novel Src inhibitors: pharmacophore-based virtual screening, molecular docking and molecular dynamics simulations. Molecules. 2020;25(18):4094. 32911607 10.3390/molecules25184094 PMC7571137

[64] Shivakumar D, Williams J, Wu Y, Damm W, Shelley J, Sherman W. Prediction of absolute solvation free energies using molecular dynamics free energy perturbation and the OPLS force field. J Chem Theory Comput. 2010;6(5):1509–1519. 26615687 10.1021/ct900587b

[65] Martyna GJ, Tobias DJ, Klein ML. Constant pressure molecular dynamics algorithms. J Chem Phys. 1994;101(5):4177–4189.

[66] Bowers KJ, Chow E, Xu H, Dror RO, Eastwood MP, Gregersen BA, Klepeis JL, Kolossvary I, Moraes MA, Sacerdoti FD, et al. Scalable algorithms for molecular dynamics simulations on commodity clusters. Paper presented at: SC'06. Proceedings of the 2006 ACM/IEEE Conference on Supercomputing. Tampa (FL): IEEE; 2006.

[67] Luty BA, Davis ME, Tironi IG, Van Gunsteren WF. A comparison of particle-particle, particle-mesh and Ewald methods for calculating electrostatic interactions in periodic molecular systems. Mol Simul. 1994;14(1):11–20.

[68] Hospital A, Goñi JR, Orozco M, Gelpí JL. Molecular dynamics simulations: advances and applications. Adv Appl Bioinform Chem. 2015;8:37. 26604800 10.2147/AABC.S70333 PMC4655909

[69] Choudhary MI, Shaikh M, Tul-Wahab A, Ur-Rahman A. In silico identification of potential inhibitors of key SARS-CoV-2 3CL hydrolase (Mpro) via molecular docking, MMGBSA predictive binding energy calculations, and molecular dynamics simulation. PLos One. 2020;15(7):e0235030. 32706783 10.1371/journal.pone.0235030 PMC7380638

