# Supplementary material for: Synthesis, kinetic studies and in-silico investigations of novel quinolinyl-iminothiazolines as alkaline phosphatase inhibitors
Source: J Enzyme Inhib Med Chem. 2023 Jan 11;38(1):2163394. doi: 10.1080/14756366.2022.2163394 (PMC9848371; doi:10.1080/14756366.2022.2163394)
Supplement: Supplemental Material [file IENZ_A_2163394_SM0115.pdf]

Supporting Information

Characterization Data

1.1.1. (*E*)-*N*-(4-(4-Bromophenyl)-3-(quinolin-3-yl)thiazol-2(3*H*)-ylidene)butyramide (6a)

Light yellow crystalline; M.P = 173 °C; yield = 77%; R_f = 0.40 (EtOAc: n-hexane, 2:8); FT-IR (ATR) in cm^{-1}, 3108 (H-C, Ar), 3064 (H-C, thiazoline), 2952, 2868 (H-C, alkyl asymmetric and symmetric), 1738 (C=O), 1588 (C=C, Ar); ^{1}H-NMR (300 MHz, $CDCl_3$); in δ (ppm), 8.95 (d, 1H, *J* = 2.1 Hz, quinolinyl-H), 8.33 (d, 1H, *J* = 8.4 Hz, quinolinyl-H), 8.21 (d, 1H, *J* = 2.4 Hz, quinolinyl-H), 7.93 (m, 2H, quinolinyl-H), 7.83 (d, 2H, *J* = 8.4 Hz, phenyl-H), 7.72 (m, 1H, quinolinyl-H), 7.39 (d, 2H, *J* = 8.4 Hz, phenyl-H), 6.48 (s, 1H, thiazoline-H), 2.36 (d, 2H, *J* = 3.6, aliphatic-H), 1.55 (sx, 2H, *J* = 7.2 Hz, aliphatic-H), 0.89 (t, 3H, *J* = 7.2 Hz, aliphatic-H); ^{13}C-NMR (75 MHz, $CDCl_3$) in δ (ppm), 173.5 (C=O), 170.8 (imine, C), 149.4, 147.2, 138.3, 135.3, 134.9,131.1, 130.8, 130.7, 129.3, 128.2, 128.0, 127.5, 126.4, 104.6 (thiazoline, C), 30.5, 20.3, 13.5. Anal. Calc. for $C_{22}H_{18}BrN_3OS$: C, 58.41; H, 4.01; N, 9.29; Found C, 58.38; H, 4.00; N, 9.25. HRMS Caled for $C_{22}H_{18}BrN_3OS$ +H : 451.0354. Found 451.0351

1.1.2. (*E*)-*N*-(4-(4-Bromophenyl)-3-(quinolin-3-yl)thiazol-2(3*H*)-ylidene)pentanamide (6b)

Brown crystalline; M.P = 173 °C; yield = 75%; R_f = 0.45 (EtOAc: n-hexane, 2:8); FT-IR (ATR) in cm^{-1}, 3059 (H-C, Ar), 2957 (H-C, thiazoline), 2928, 2858 (H-C, alkyl asymmetric and symmetric), 1738 (C=O), 1589 (C=C, Ar); ^{1}H-NMR (300 MHz, $CDCl_3$); in δ (ppm), 8.93 (d, 1H, *J* = 2.1 Hz, quinolinyl-H), 8.32 (d, 1H, *J* = 8.4 Hz, quinolinyl-H), 8.23 (d, 1H, *J* = 2.4 Hz, quinolinyl-H), 7.91 (m, 2H, quinolinyl-H), 7.82 (d, 2H, *J* = 8.4 Hz, phenyl-H), 7.70 (m, 1H, quinolinyl-H), 7.41 (d, 2H, *J* = 8.4 Hz, phenyl-H), 6.45 (s, 1H, thiazoline-H), 2.36 (t, 2H, *J* = 7.2 Hz, aliphatic-H), 1.53 (m, 2H, aliphatic-H), 1.2 (m, 2H, aliphatic-H), 0.90 (t, 3H, *J* = 7.5 Hz, aliphatic-H); ^{13}C-NMR (75 MHz, $CDCl_3$) in δ (ppm), 172.5 (C=O), 171.0 (imine, C), 149.2, 148.1, 137.3, 134.6, 133.8,130.9, 129.8, 129.4, 128.5, 127.5, 127.0, 126.9, 126.2, 105.0 (thiazoline, C), 31.2, 22.5, 19.2, 13.2. Anal. Calc. for $C_{23}H_{20}BrN_3OS$: C, 59.23; H, 4.32; N, 9.01; Found: C, 59.21; H, 4.31; N, 9.00. HRMS Caled for $C_{23}H_{20}BrN_3OS$ +H : 465.0510. Found 465.0507

Information Classification: General

1.1.3. (*E*)-*N*-(4-(4-Bromophenyl)-3-(quinolin-3-yl)thiazol-2(3*H*)-ylidene)hexanamide (6c)

Off-white powder; M.P = 181-182 ^{o}C; yield = 68%; R_f = 0.48 (EtOAc: n-hexane, 2:8); FT-IR (ATR) in cm^{-1}, 3102 (H-C, Ar), 3050 (H-C, thiazoline), 2913, 2850 (H-C, alkyl asymmetric and symmetric), 1736 (C=O), 1588 (C=C, Ar); ^{1}H-NMR (300 MHz, $CDCl_3$); in δ (ppm), 8.94 (d, 1H, *J* = 2.1 Hz, quinolinyl-H), 8.30 (d, 1H, *J* = 8.4 Hz quinolinyl-H), 8.19 (d, 1H, *J* = 2.1 Hz, quinolinyl-H), 7.93 (m, 2H, quinolinyl-H), 7.84 (d, 2H, *J* = 8.4 Hz, phenyl-H), 7.72 (m, 1H, quinolinyl-H), 7.40 (d, 2H, *J* = 8.4 Hz, phenyl-H), 6.49 (s, 1H, thiazoline-H), 2.39 (m, 2H, aliphatic-H), 1.53 (m, 2H, aliphatic-H), 1.22 (m, 4H, aliphatic-H), 0.82 (t, 3H, *J* = 7.5 Hz aliphatic-H); ^{13}C-NMR (75 MHz, $CDCl_3$) in δ (ppm), 173.6 (C=O), 170.8 (imine, C), 149.6, 147.6, 138.6, 135.3, 134.5,131.1, 131.0, 130.8, 130.7, 129.6, 128.1, 127.9, 127.5, 126.4, 104.5 (thiazoline, C), 31.0, 28.5, 26,6 22.2, 13.7. Anal. Calc. for $C_{24}H_{22}BrN_3OS$: C, 60.00; H, 4.62; N, 8.75; Found: C, 59.98; H, 4.59; N, 8.71. . HRMS Caled for $C_{24}H_{22}BrN_3OS$ +H : 479.0667. Found 479.0664

1.1.4. (*E*)-*N*-(4-(4-Bromophenyl)-3-(quinolin-3-yl)thiazol-2(3*H*)-ylidene)heptanamide (6d)

Light yellow powder; M.P = 188-190 ^{o}C; yield = 70; R_f = 0.50 (EtOAc: n-hexane, 2:8); FT-IR (ATR) in cm^{-1}, 3098 (H-C, Ar), 3012 (H-C, thiazoline), 2958, 2860 (H-C, alkyl asymmetric and symmetric), 1715 (C=O), 1580 (C=C, Ar); ^{1}H-NMR (300 MHz, $CDCl_3$); in δ (ppm), 8.89 (d, 1H, *J* = 2.4 Hz, quinolinyl-H), 8.27 (d, 1H, *J* = 8.4 Hz, quinolinyl-H), 8.19 (d, 1H, *J* = 2.4 Hz, quinolinyl-H), 7.88 (m, 2H, quinolinyl-H), 7.80 (d, 2H, *J* = 8.4 Hz, phenyl-H), 7.67 (m, 1H, quinolinyl-H), 7.40 (d, 2H, *J* = 8.4 Hz, phenyl-H), 6.43 (s, 1H, thiazoline-H), 2.38 (m, 2H, aliphatic-H), 1.55 (m, 2H, aliphatic-H), 1.30 (m, 6H, aliphatic-H), 0.92 (t, 3H, *J* = 7.2 Hz, aliphatic-H); ^{13}C-NMR(75 MHz, $CDCl_3$) in δ (ppm), 172.3 (C=O), 170.8 (imine, C), 149.0, 147.4, 137.1, 134.2, 132.8,130.4, 129.3, 128.6, 128.2, 127.9, 127.1, 126.5, 126.0, 104.3 (thiazoline, C), 31.2, 29.7, 28.2, 24.6, 22.2, 13.5. Anal. Calc. for $C_{25}H_{24}BrN_3OS$: C, 60.73; H, 4.89; N, 8.50; Found: C, 60.69; H, 4.87; N, 8.47. HRMS Caled for $C_{25}H_{24}BrN_3OS$ +H : 493.0823. Found 493.0823

Information Classification: General

1.1.5. (*E*)-*N*-(4-(4-Bromophenyl)-3-(quinolin-3-yl)thiazol-2(3*H*)-ylidene)octanamide (6e)

Light yellow powder; M.P = 192 ^oC; yield = 71%; R_f = 0.52 (EtOAc: n-hexane, 2:8); FT-IR (ATR) in cm^{-1}, 3112 (H-C, Ar), 3055 (H-C, thiazoline), 2920, 2843 (H-C, alkyl asymmetric and symmetric), 1702 (C=O), 1592 (C=C, Ar); ^{1}H-NMR (300 MHz, $CDCl_3$); in δ (ppm), 8.93 (d, 1H, *J* = 2.1 Hz, quinolinyl-H), 8.31 (d, 1H, *J* = 8.4 Hz, quinolinyl-H), 8.20 (d, 1H, *J* = 2.1 Hz, quinolinyl-H), 7.94 (m, 2H, quinolinyl-H), 7.86 (d, 2H, *J* = 8.4 Hz, phenyl-H), 7.74(m, 1H, quinolinyl-H), 7.42 (d, 2H, *J* = 8.4 Hz, phenyl-H), 6.51 (s, 1H, thiazoline-H), 2.63 (m, 2H, aliphatic-H), 1.72 (m, 2H, aliphatic-H), 1.35 (m, 8H, aliphatic-H), 0.89 (t, 3H, *J* = 7.5 Hz, aliphatic-H); ^{13}C-NMR (75 MHz, $CDCl_3$) in δ (ppm), 172.0 (C=O), 170.7 (imine, C), 148.4, 147.3, 138.1, 134.7, 134.0,130.9, 130.3, 129.9, 129.6, 128.0, 127.8, 127.4, 126.3, 104.9 (thiazoline, C), 31.0, 29.7, 28.5, 26,6, 24.5, 22.2, 13.7. Anal. Calc. for $C_{26}H_{26}BrN_3OS$: C, 61.41; H, 5.15; N, 8.26; Found: C, 61.39; H, 5.12; N, 8.25. HRMS Caled for $C_{26}H_{26}BrN_3OS$ +H : 507.0980 Found 507.0980

1.1.6. (*E*)-*N*-(4-(4-Bromophenyl)-3-(quinolin-3-yl)thiazol-2(3*H*)-ylidene)decanamide (6f)

Yellow powder; M.P = 180 ^oC; yield = 73%; R_f = 0.53 (EtOAc: n-hexane, 2:8); FT-IR (ATR) in cm^{-1}, 3105 (H-C, Ar), 3038 (H-C, thiazoline), 2930, 2865 (H-C, alkyl asymmetric and symmetric), 1705 (C=O), 1583 (C=C, Ar); ^{1}H-NMR (300 MHz, $CDCl_3$); in δ (ppm), 8.96 (d, 1H, *J* = 2.4 Hz, quinolinyl-H), 8.33 (d, 1H, *J* = 2.4 Hz, quinolinyl-H), 8.21 (d, 1H, *J* = 8.4 Hz, quinolinyl-H), 7.95 (m, 2H, quinolinyl-H), 7.87 (d, 2H, *J* = 8.4 Hz, phenyl-H), 7.76 (m, 1H, quinolinyl-H), 7.43 (d, 2H, *J* = 8.4 Hz, phenyl-H), 6.53 (s, 1H, thiazoline-H), 2.44 (m, 2H, aliphatic-H), 1.73 (m, 2H, aliphatic-H), 1.34 (m, 12H, aliphatic-H), 0.89 (t, 3H, *J* = 7.2 Hz, aliphatic-H); ^{13}C-NMR (75 MHz, $CDCl_3$) in δ (ppm), 172.6 (C=O), 170.7 (imine, C), 148.9, 147.6, 137.4, 135.1, 133.7,131.5, 131.1, 130.8, 130.5, 129.2, 128.1, 127.8, 127.6, 125.4, 105.3 (thiazoline, C), 37.3, 31.8, 29.4, 29.3, 29.2, 29.0 24.7, 22.6, 14.1. Anal. Calc. for $C_{28}H_{30}BrN_3OS$: C, 62.68; H, 5.64; N, 7.83; Found: C, 62.65; H, 5.62; N, 7.80. HRMS Caled for $C_{28}H_{30}BrN_3OS$ +H : 535.1293 Found 535.1290

Information Classification: General

1.1.7. (*E*)-*N*-(4-(4-Bromophenyl)-3-(quinolin-3-yl)thiazol-2(3*H*)-ylidene)benzamide (6g)

Yellow powder; M.P = 217-220 °C; yield = 69%; R_f = 0.43 (EtOAc: n-hexane, 2:8); FT-IR (ATR) in cm^{-1}, 3112 (H-C, Ar), 3062 (H-C, thiazoline), 1701 (C=O), 1588 (C=C, Ar); ^{1}H-NMR (300 MHz, $CDCl_3$); in δ (ppm), 8.84 (d, 1H, *J* = 2.4 Hz, quinolinyl-H), 8.27 (d, 1H, *J* = 8.4 Hz, quinolinyl-H), 8.17 (d, 1H, *J* = 2.4 Hz, quinolinyl-H), 7.94 (m, 2H, quinolinyl-H), 7.89 (m, 2H, Ar-H) 7.84 (d, 2H, *J* = 8.4 Hz, phenyl-H), 7.79 (m, 2H, Ar-H), 7.70 (m, 1H, quinolinyl-H), 7.61 (m, 1H, Ar-H), 7.39 (d, 2H, *J* = 8.4 Hz, phenyl-H), 6.47 (s, 1H, thiazoline-H); ^{13}C-NMR (75 MHz, $CDCl_3$) in δ (ppm), 172.6 (C=O), 169.8 (imine, C), 149.9, 146.7, 137.5, 136.1, 135.2 134.3, 134.1, 131.1, 131.0, 130.8, 130.7, 129.9, 129.6, 129.2, 128.1, 127.9, 127.3, 126.2, 105.5 (thiazoline, C). Anal. Calc. for $C_{25}H_{16}BrN_3OS$: C, 61.73; H, 3.32; N, 8.64; Found: C, 61.71; H, 3.29; N, 8.61. HRMS Caled for $C_{25}H_{16}BrN_3OS$+H : 485.0197 Found 485.0194

1.1.8. (*E*)-*N*-(4-(4-Bromophenyl)-3-(quinolin-3-yl)thiazol-2(3*H*)-ylidene)-4-methylbenzamide (6h)

Yellow powder; M.P = 103-105 °C; yield = 68%; R_f = 0.45 (EtOAc: n-hexane, 2:8); FT-IR (ATR) in cm^{-1}, 3114 (H-C, Ar), 3041 (H-C, thiazoline), 2913, 2850 (H-C, alkyl asymmetric and symmetric), 1709 (C=O), 1570 (C=C, Ar); ^{1}H-NMR (300 MHz, $CDCl_3$); in δ (ppm), 8.86 (d, 1H, *J* = 2.4 Hz, quinolinyl-H), 8.23 (d, 1H, *J* = 8.4 Hz, quinolinyl-H), 8.14 (d, 1H, *J* = 2.4 Hz, quinolinyl-H), 7.92 (m, 2H, quinolinyl-H), 7.81 (d, 2H, *J* = 8.4 Hz, phenyl-H), 7.77 (d, 2H, *J* = 7.8 Hz, Ar-H) 7.70 (m, 1H, quinolinyl-H), 7.48 (d, 2H, *J* = 7.8 Hz, Ar-H), 7.39 (d, 2H, *J* = 8.4 Hz, phenyl-H), 6.39 (s, 1H, thiazoline-H), 2.19 (s, 3H, aliphatic-H); ^{13}C-NMR (75 MHz, $CDCl_3$) in δ (ppm), 171.2 (C=O), 168.9 (imine, C), 150.1, 147.7, 144.1, 137.5, 135.3, 134.5, 132.3, 131.1, 130.9, 130.6, 130.2, 130.0, 129.7, 129.0, 128.3, 126.9, 126.3, 125.4, 106.2 (thiazoline, C), 22.2. Anal. Calc. for $C_{26}H_{18}BrN_3OS$: C, 62.40; H, 3.63; N, 8.40; Found: C, 62.38; H, 3.60; N, 8.37. HRMS Caled for $C_{26}H_{18}BrN_3OS$ +H : 499.0354 Found 499.0351

1.1.9. (*E*)-*N*-(4-(4-Bromophenyl)-3-(quinolin-3-yl)thiazol-2(3*H*)-ylidene)-2,4-dichlorobenzamide (6i)

Information Classification: General

Off-white powder; M.P = 198 °C; yield = 64%; R_f = 0.32 (EtOAc: n-hexane, 2:8); FT-IR (ATR) in cm^{-1}, 3115 (H-C, Ar), 3038 (H-C, thiazoline), 1679 (C=O), 1590 (C=C, Ar); ^{1}H-NMR (300 MHz, $CDCl_3$); in δ (ppm), 8.79 (d, 1H, *J* = 2.4 Hz, quinolinyl-H), 8.29 (d, 1H, *J* = 8.4 Hz, quinolinyl-H), 8.19 (d, 1H, *J* = 2.4 Hz, quinolinyl-H), 7.93 (m, 2H, quinolinyl-H), 7.88 (m, 2H, Ar-H) 7.82 (d, 2H, *J* = 8.4 Hz, phenyl-H), 7.79 (m, 1H, Ar-H), 7.69 (m, 2H, quinolinyl, Ar-H), 7.61 (d, 1H, Ar-H), 7.41 (d, 2H, *J* = 8.4 Hz, phenyl-H), 6.39 (s, 1H, thiazoline-H); ^{13}C-NMR (75 MHz, $CDCl_3$) in δ (ppm), 173.4 (C=O), 171.3 (imine, C), 148.9, 147.6, 137.6, 136.3, 135.5 134.7, 134.2, 132.8, 131.9, 131.4, 130.7, 130.2, 129.9, 129.6, 128.7, 128.1, 127.9, 127.5, 126.2, 104.3 (thiazoline, C). Anal. Calc. for $C_{25}H_{14}BrCl_2N_3OS$: C, 54.08; H, 2.54; N, 7.57; Found: C, 54.05; H, 2.52; N, 7.55. HRMS Caled for $C_{25}H_{14}BrCl_2N_3OS$ +H : 552.9418 Found 552.9415

(*E*)-*N*-(4-(4-Bromophenyl)-3-(quinolin-3-yl)thiazol-2(3*H*)-ylidene)-3,5-dinitrobenzamide (6j)

Orange powder; M.P = 205 °C; yield = 62%; R_f = 0.27 (EtOAc: n-hexane, 2:8); FT-IR (ATR) in cm^{-1}, 3080 (H-C, Ar), 3004 (H-C, thiazoline), 1680 (C=O), 1584 (C=C, Ar); ^{1}H-NMR (300 MHz, $CDCl_3$); in δ (ppm), 8.81 (s, 1H, Ar-H), 8.77 (d, 1H, *J* = 2.1 Hz, quinolinyl-H), 8.60 (s, 2H, Ar-H), 8.19 (d, 1H, *J* = 8.4 Hz, quinolinyl-H), 8.07 (d, 1H, *J* = 2.1 Hz, quinolinyl-H), 7.96 (m, 2H, quinolinyl-H), 7.85 (d, 2H, *J* = 8.4 Hz, phenyl-H), 7.73(m, 1H, quinolinyl-H), 7.37 (d, 2H, *J* = 8.4 Hz, phenyl-H), 6.51 (s, 1H, thiazoline-H); ^{13}C-NMR (75 MHz, $CDCl_3$) in δ (ppm), 173.6 (C=O), 170.8 (imine, C), 151.1, 148.4, 147.9, 138.5, 137.2, 135.4, 134.9, 131.9, 130.7, 129.0, 128.7, 128.0, 126.4, 123.3, 103.6 (thiazoline, C). Anal. Calc. for $C_{25}H_{14}BrN_5O_5S$: C, 52.10; H, 2.45; N, 12.15; Found: C, 52.07; H, 2.42; N, 12.13. HRMS Caled for $C_{25}H_{14}BrN_5O_5S$ +H 574.9899 Found 574.9895

Information Classification: General

NMR Spectra

(*E*)-*N*-(4-(4-Bromophenyl)-3-(quinolin-3-yl)thiazol-2(3*H*)-ylidene)butyramide (6a)

^{1}H-NMR

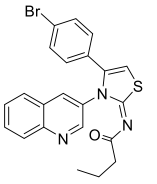

6a

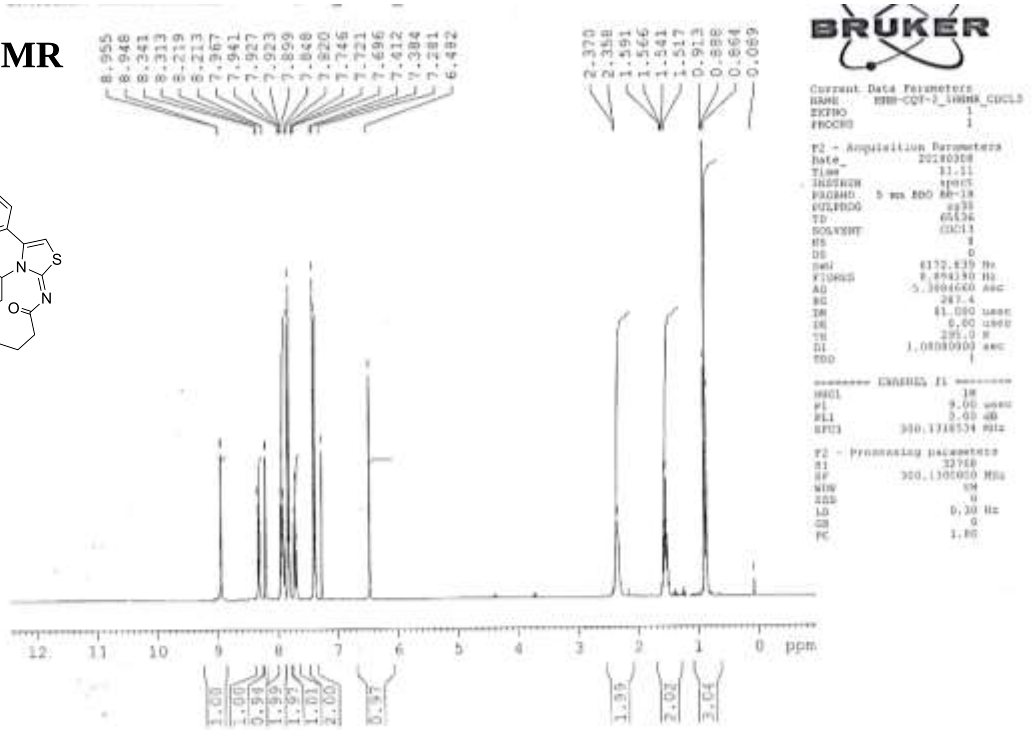

^{13}C-NMR

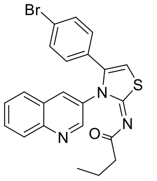

6a

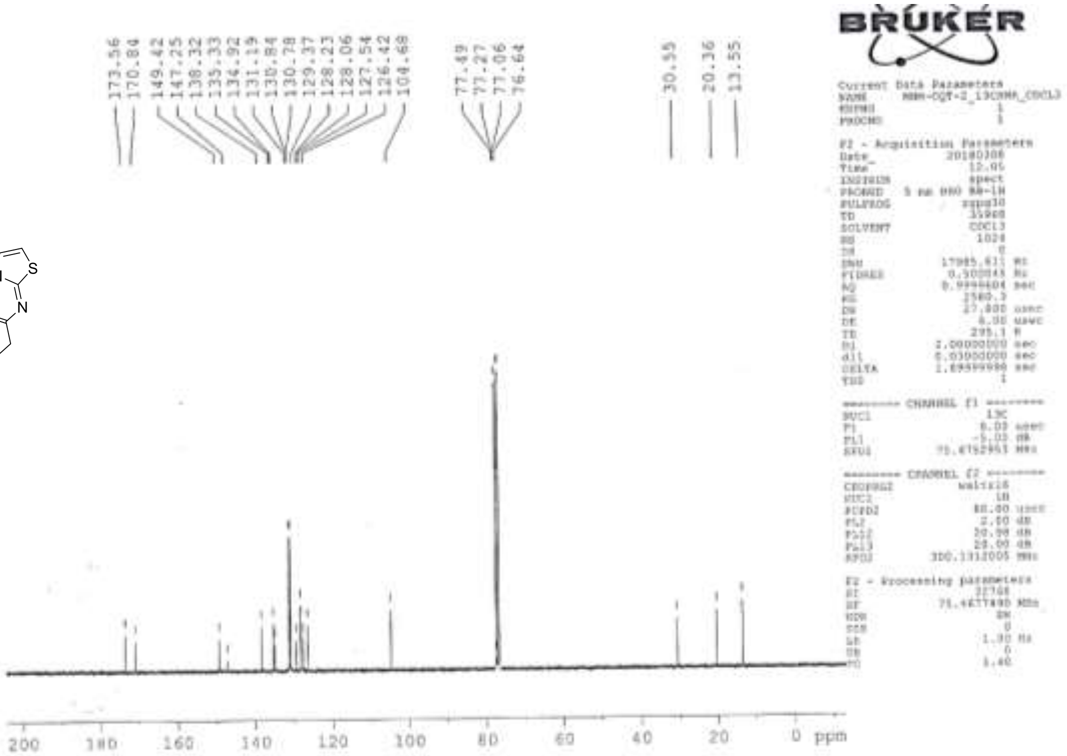

(*E*)-*N*-(4-(4-Bromophenyl)-3-(quinolin-3-yl)thiazol-2(3*H*)-ylidene)pentanamide (6b)

^{1}H-NMR

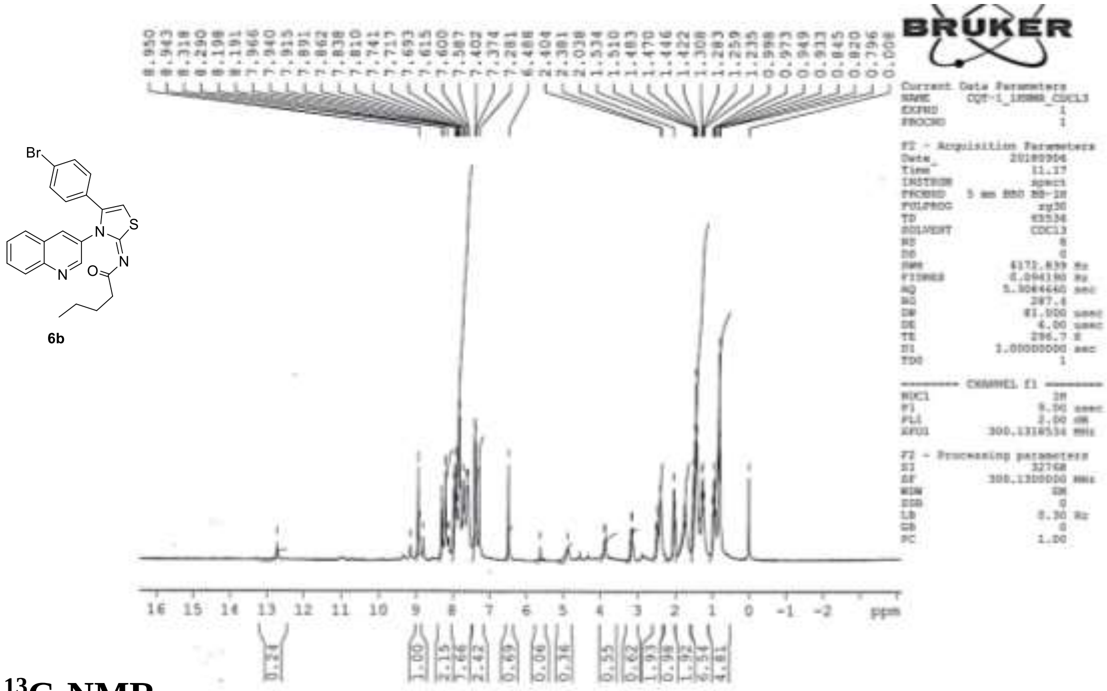

^{13}C-NMR

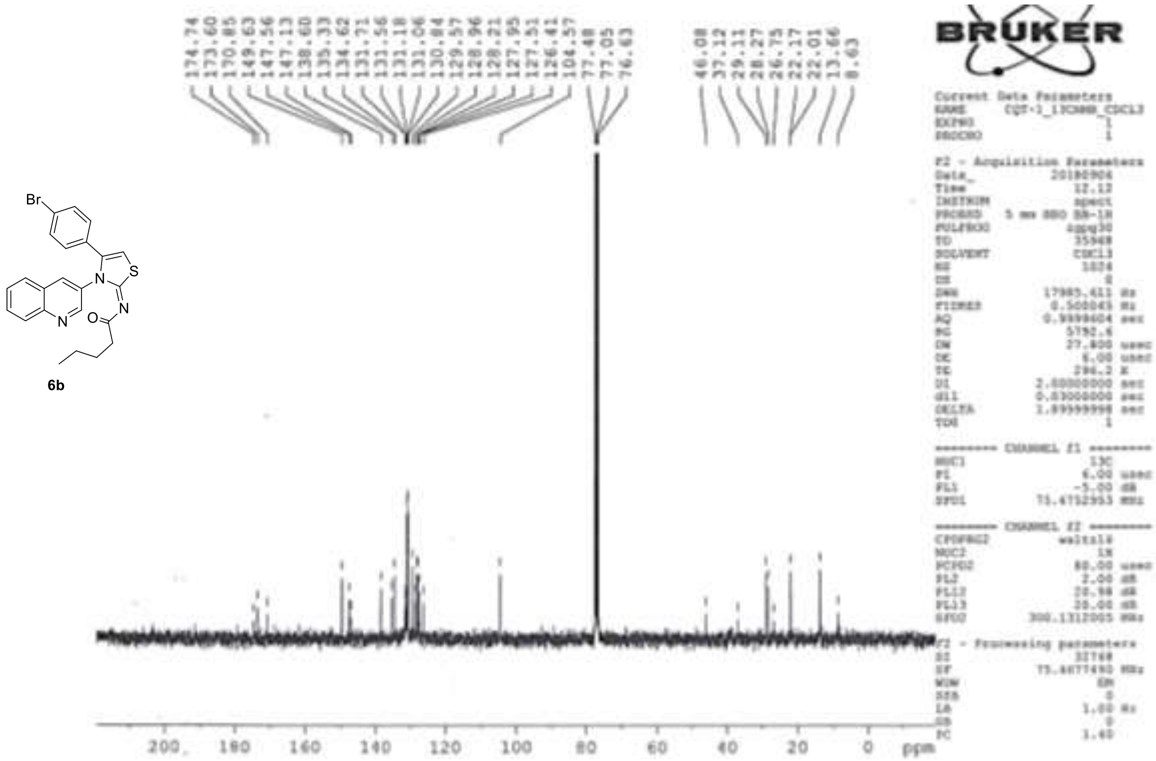

(*E*)-*N*-(4-(4-Bromophenyl)-3-(quinolin-3-yl)thiazol-2(3*H*)-ylidene)hexanamide (6c)

^{1}H-NMR

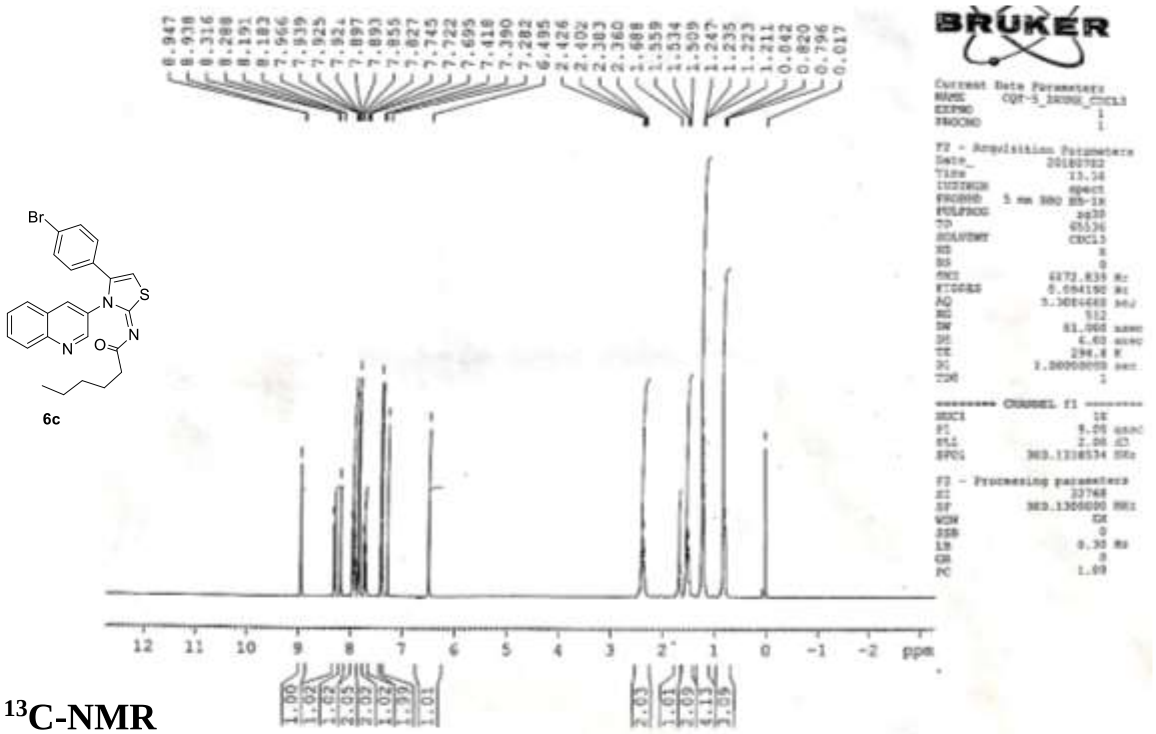

^{13}C-NMR

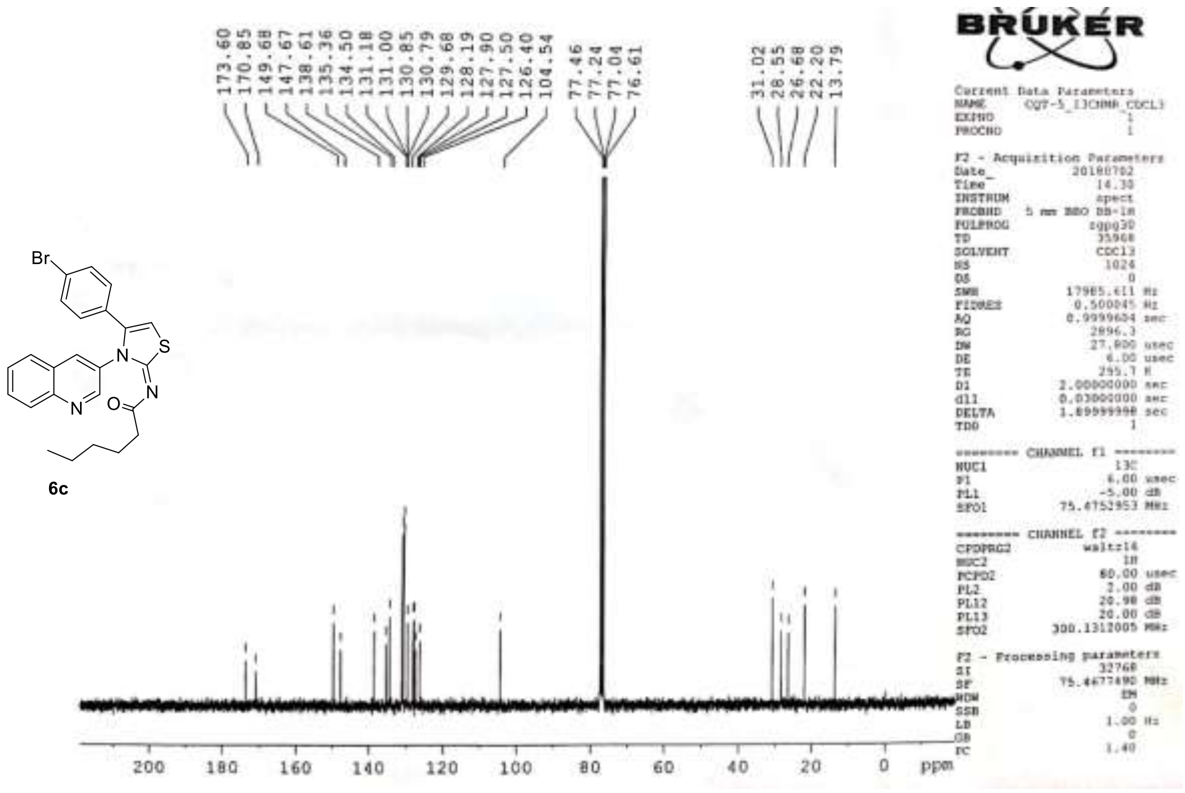

1.1.10. (*E*)-*N*-(4-(4-Bromophenyl)-3-(quinolin-3-yl)thiazol-2(3*H*)-ylidene)benzamide (6g)

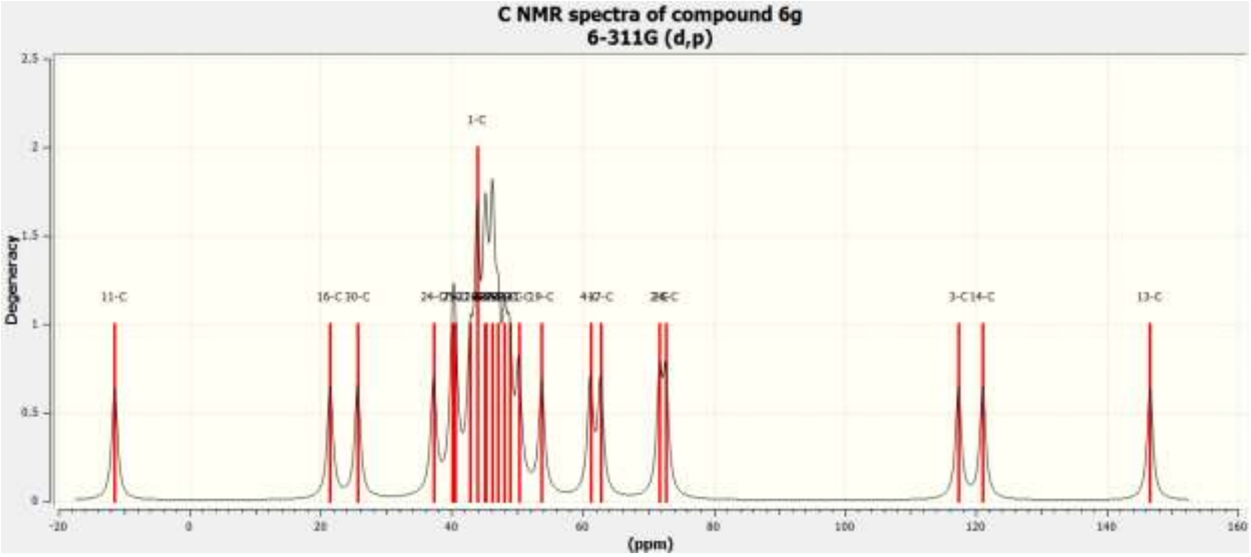

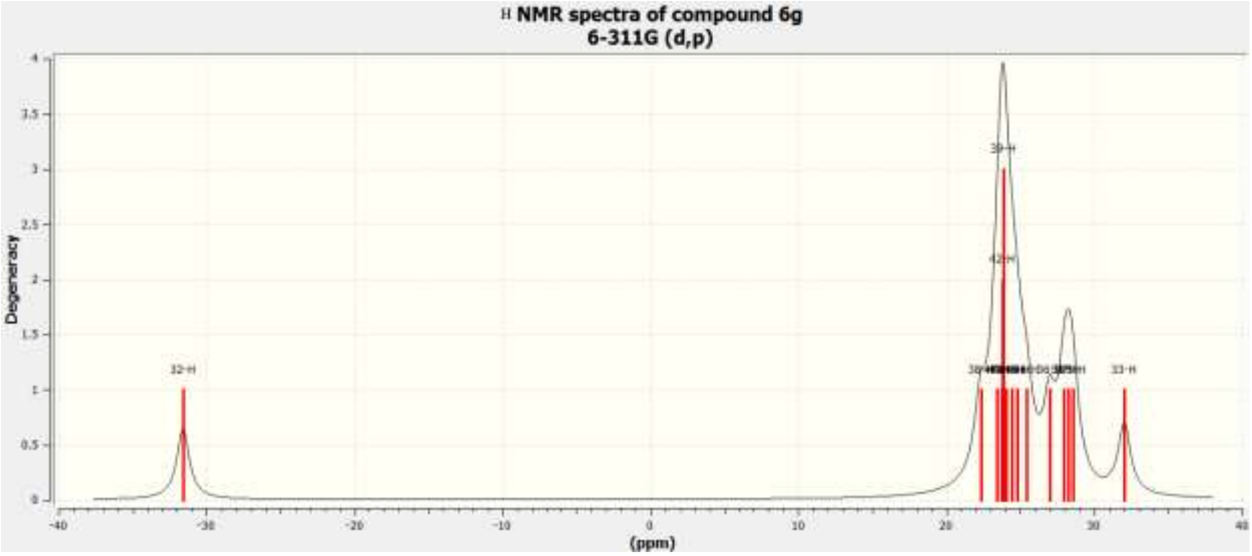

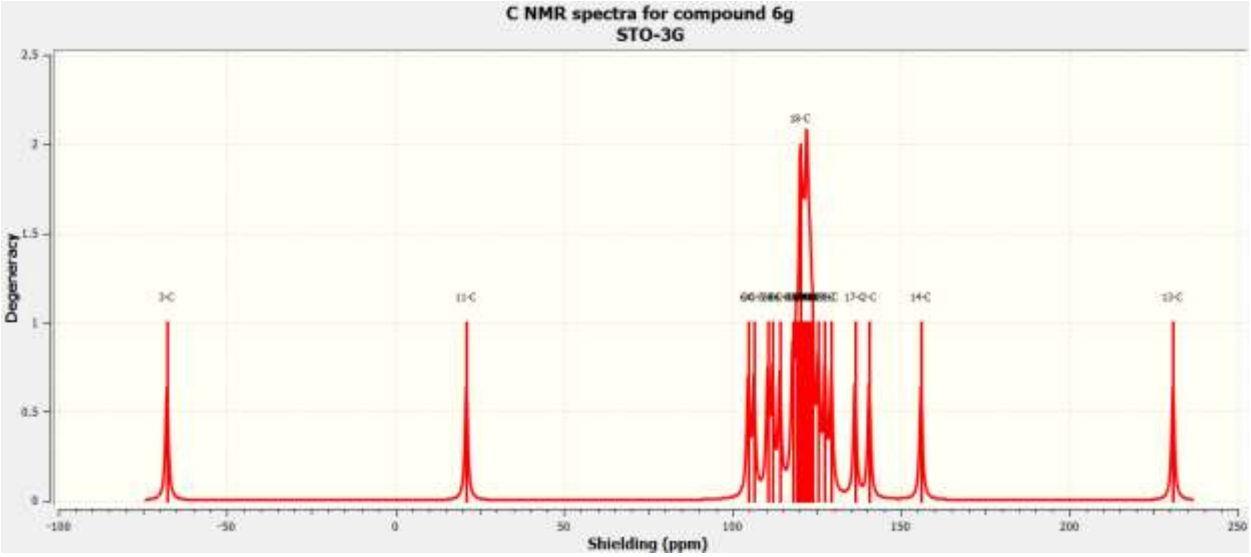

Information Classification: General

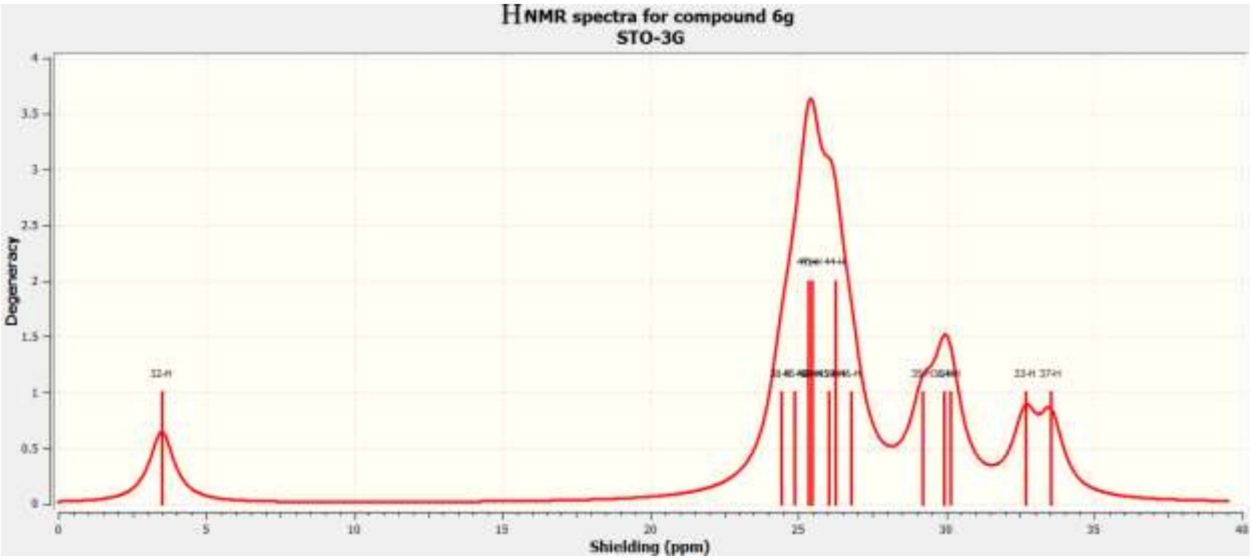

(*E*)-*N*-(4-(4-Bromophenyl)-3-(quinolin-3-yl)thiazol-2(3*H*)-ylidene)-3,5-dinitrobenzamide (6j)

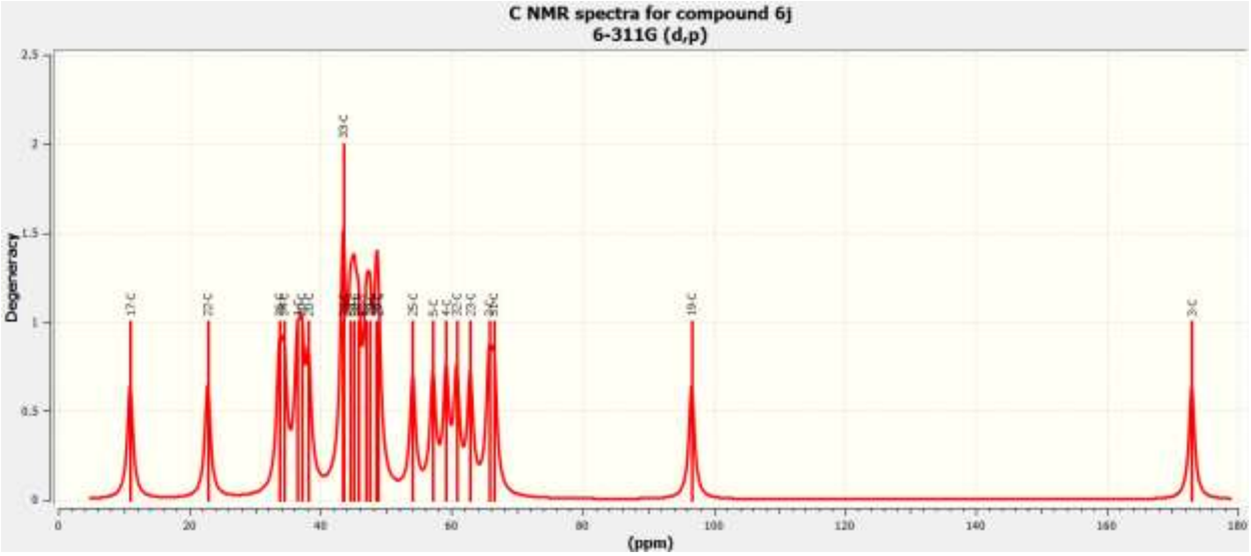

Information Classification: General

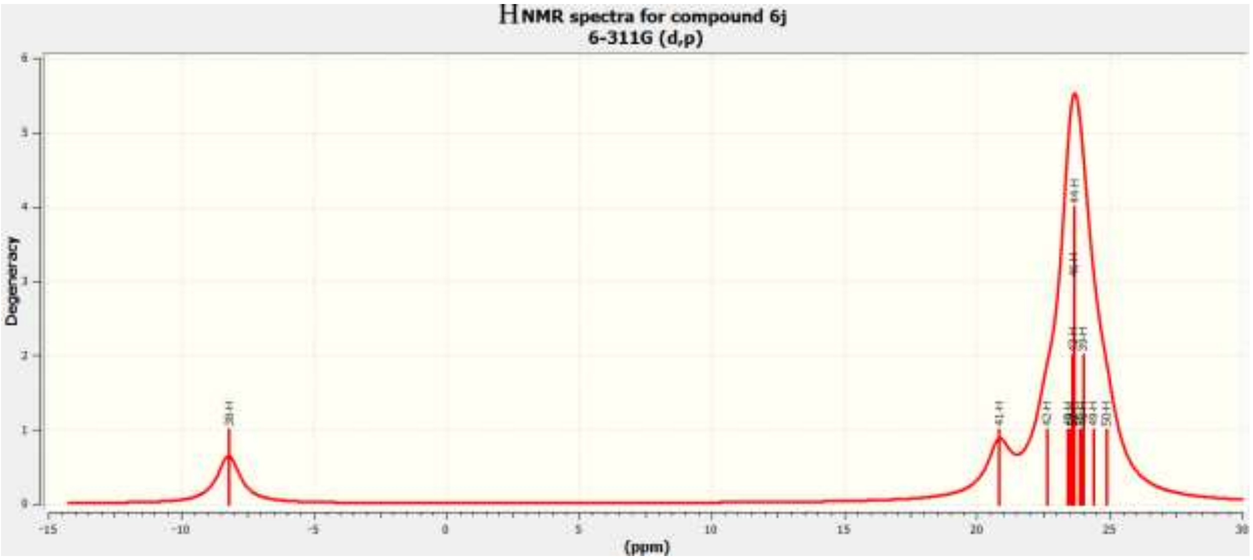

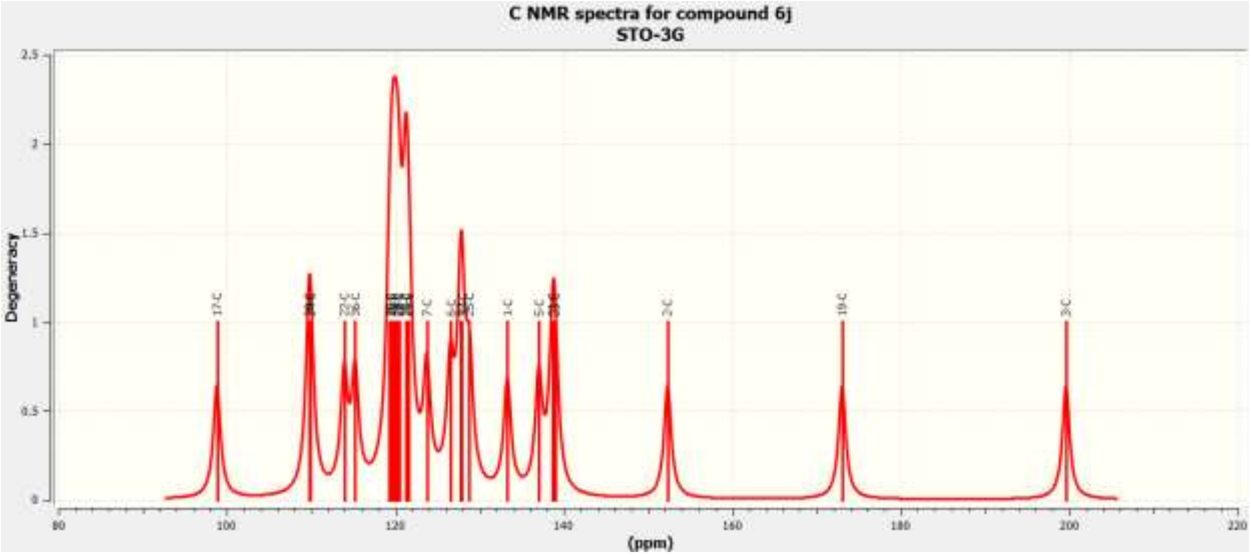

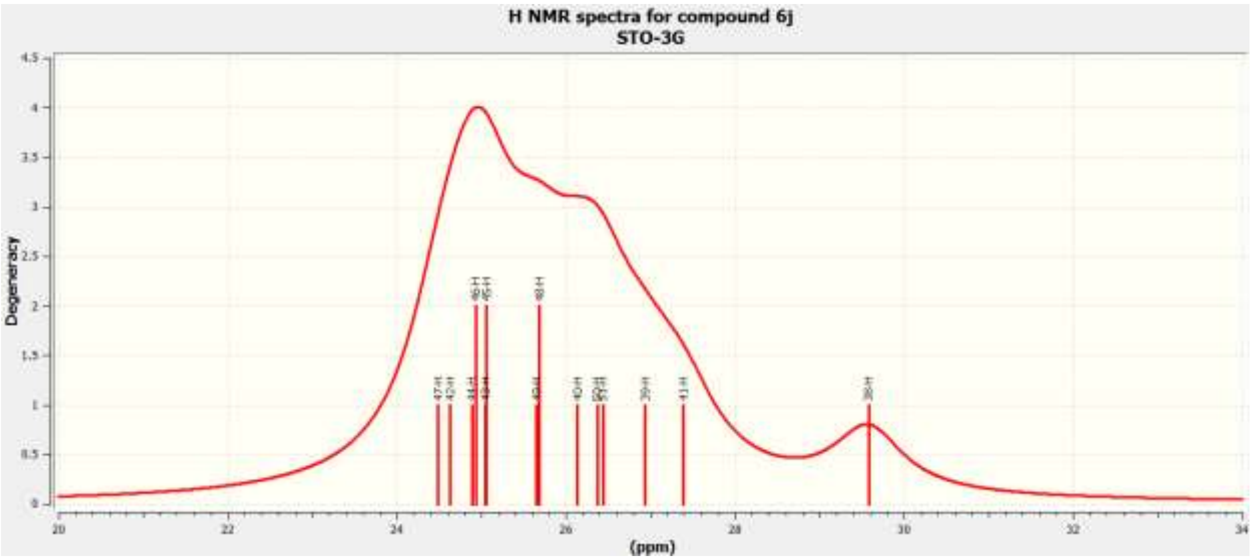

Information Classification: General

FT-IR

(*E*)-*N*-(4-(4-Bromophenyl)-3-(quinolin-3-yl)thiazol-2(3*H*)-ylidene)butyramide (6a)

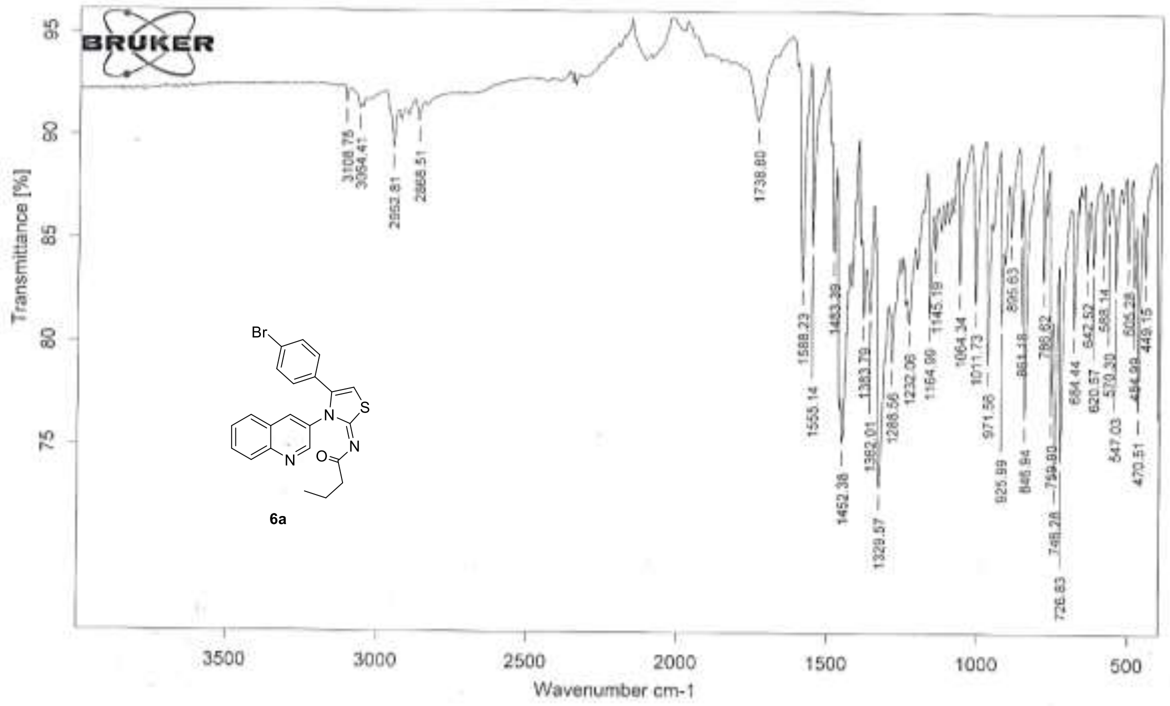

(*E*)-*N*-(4-(4-Bromophenyl)-3-(quinolin-3-yl)thiazol-2(3*H*)-ylidene)pentanamide (6b)

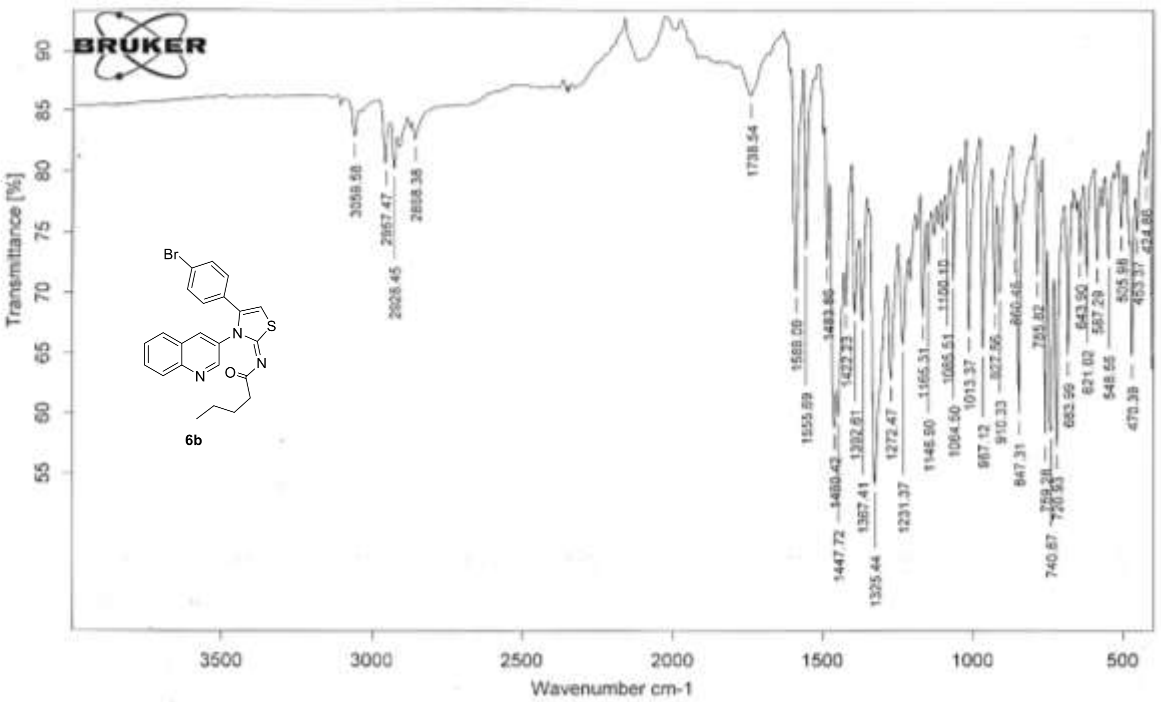

Information Classification: General

(*E*)-*N*-(4-(4-Bromophenyl)-3-(quinolin-3-yl)thiazol-2(3*H*)-ylidene)hexanamide (6c)

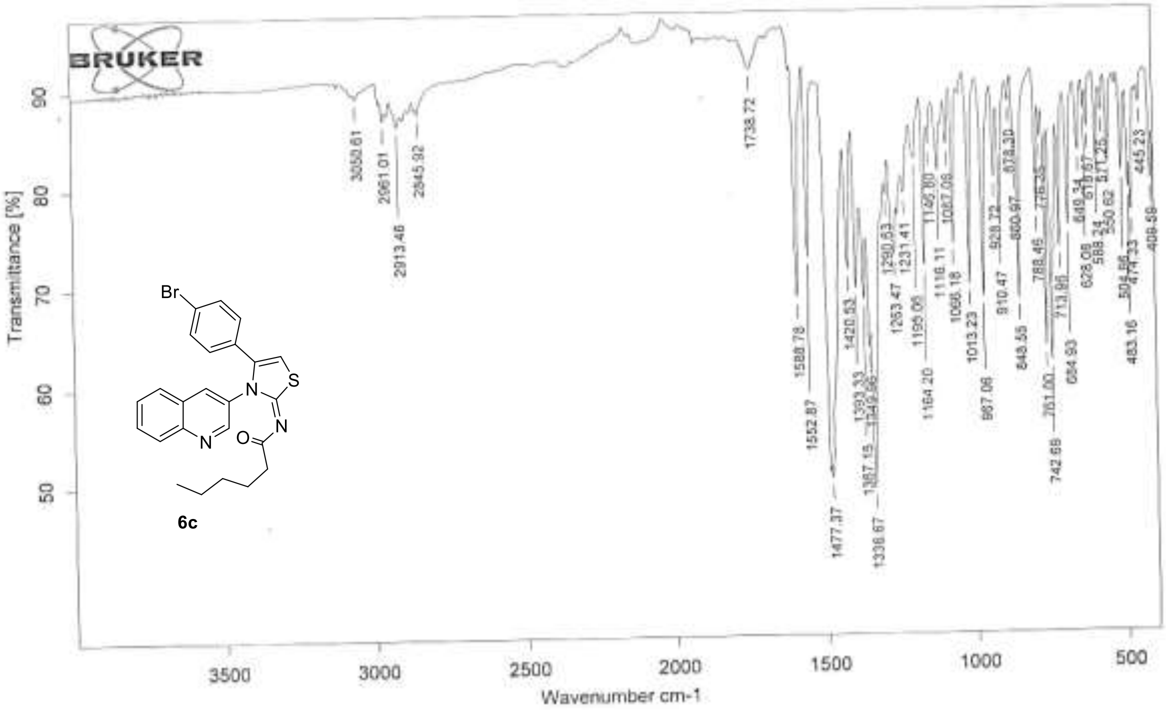

(*E*)-*N*-(4-(4-Bromophenyl)-3-(quinolin-3-yl)thiazol-2(3*H*)-ylidene)heptanamide (6d)

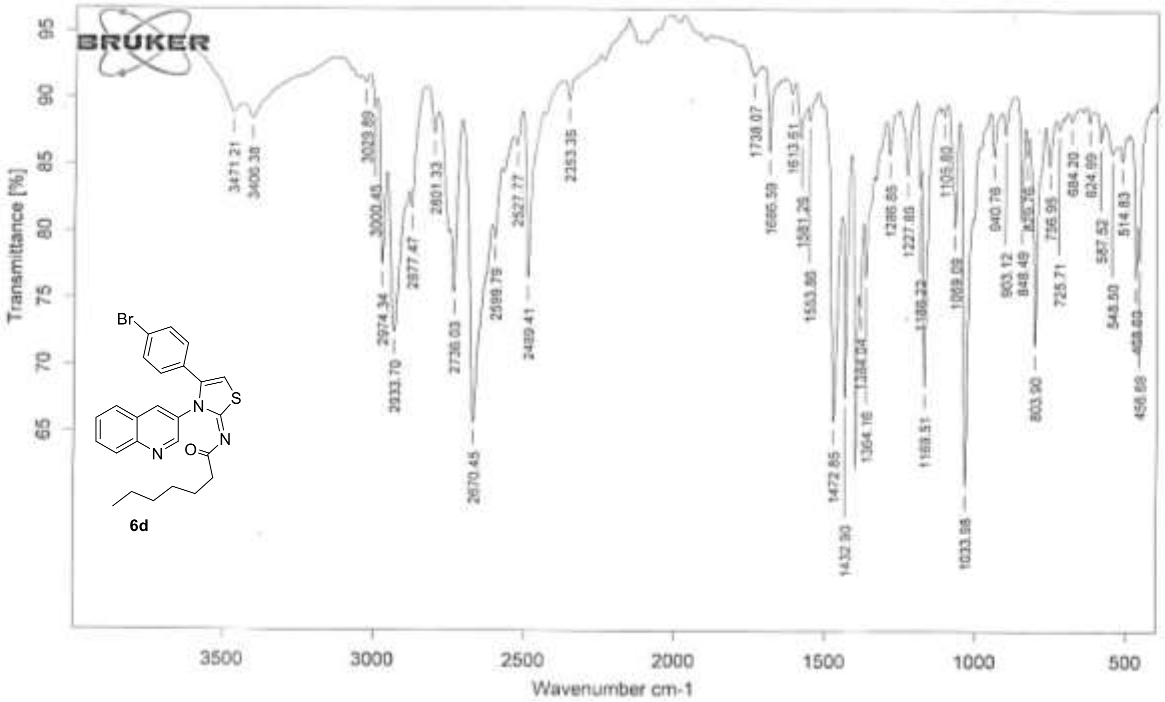

(*E*)-*N*-(4-(4-Bromophenyl)-3-(quinolin-3-yl)thiazol-2(3*H*)-ylidene)decanamide (6f)

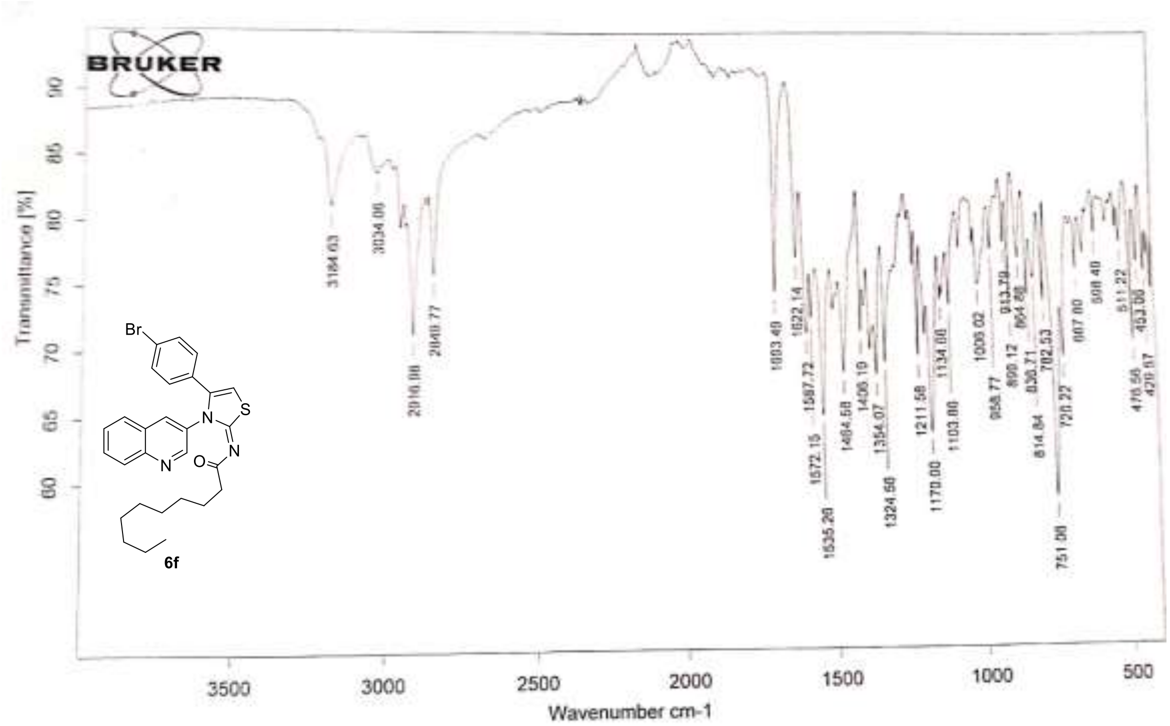

(*E*)-*N*-(4-(4-Bromophenyl)-3-(quinolin-3-yl)thiazol-2(3*H*)-ylidene)benzamide (6g)

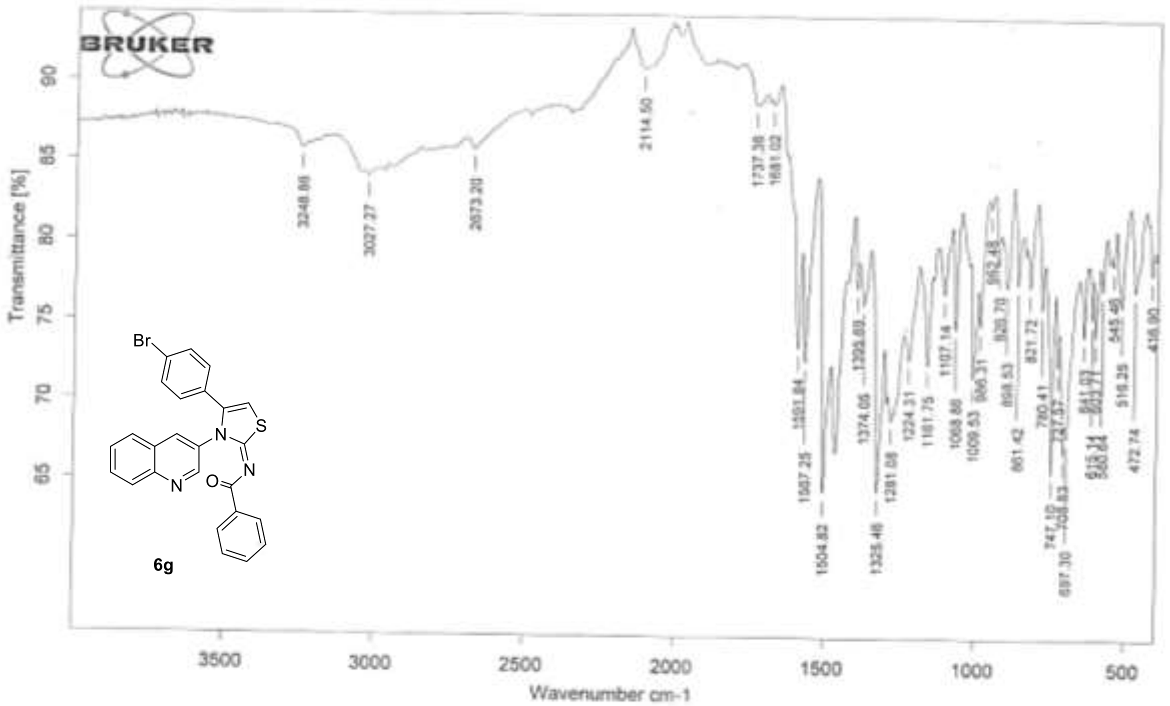

Information Classification: General

(*E*)-*N*-(4-(4-Bromophenyl)-3-(quinolin-3-yl)thiazol-2(3*H*)-ylidene)-4-methylbenzamide (6h)

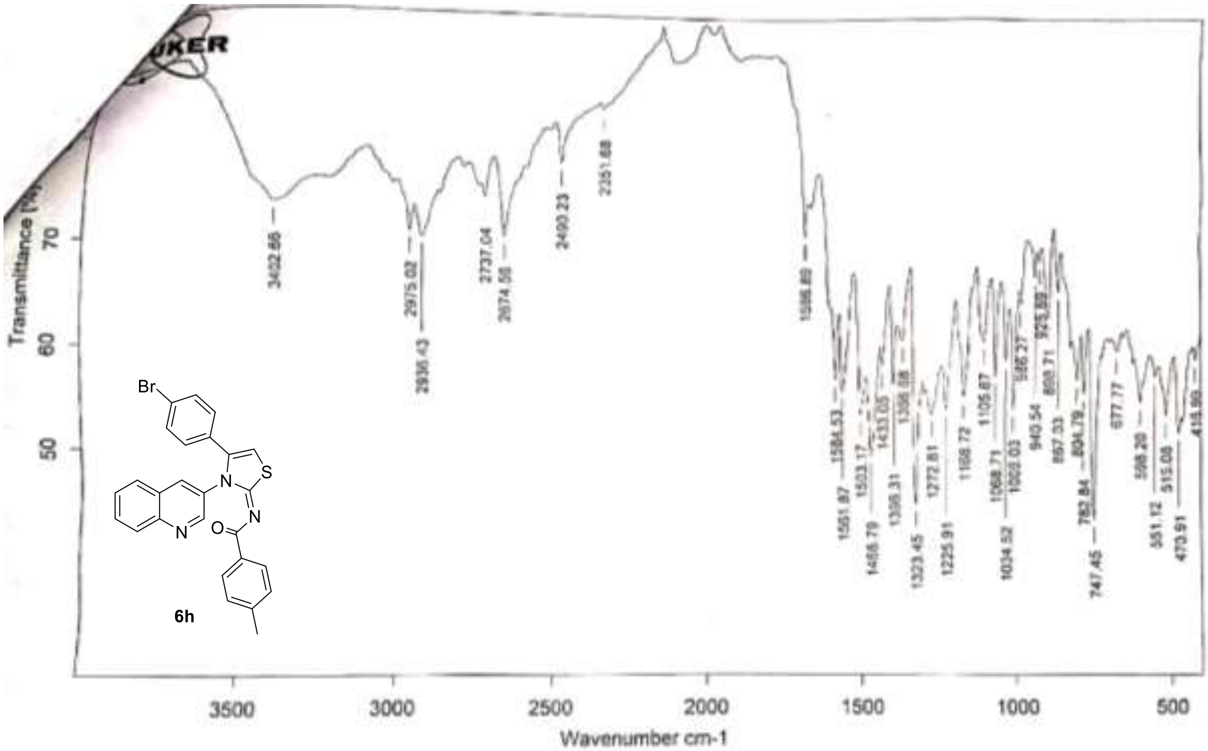

(*E*)-*N*-(4-(4-Bromophenyl)-3-(quinolin-3-yl)thiazol-2(3*H*)-ylidene)-2,4-dichlorobenzamide (6i)

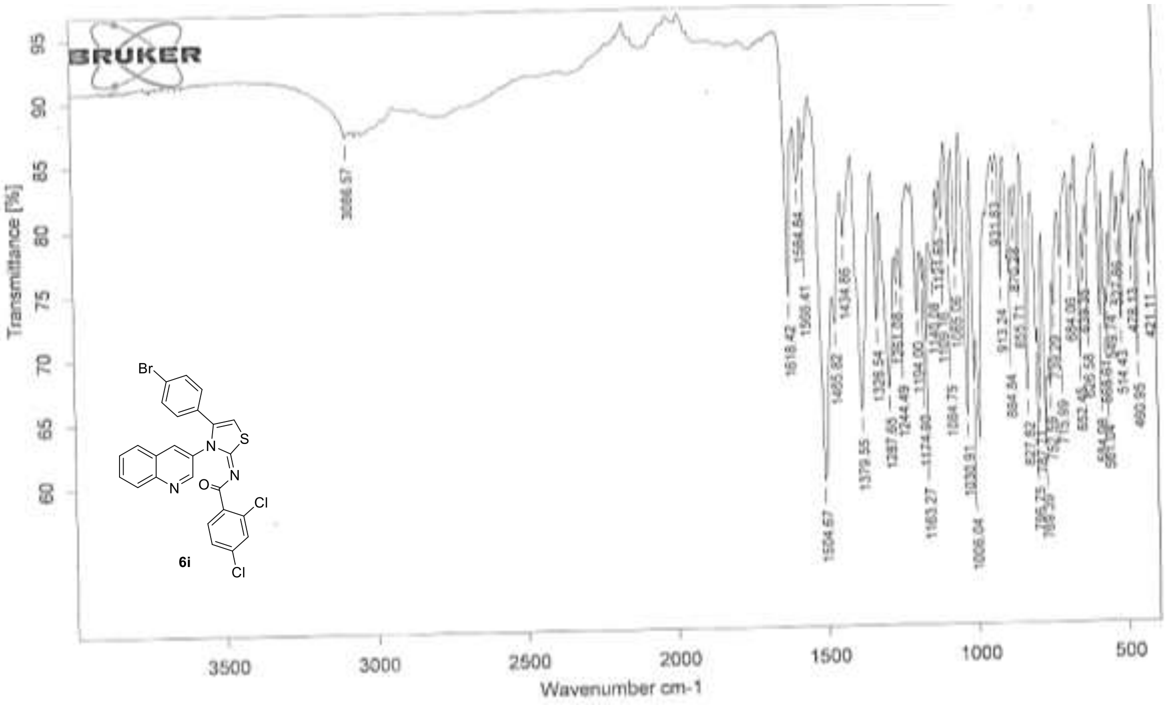

Information Classification: General

(*E*)-*N*-(4-(4-Bromophenyl)-3-(quinolin-3-yl)thiazol-2(3*H*)-ylidene)-3,5-dinitrobenzamide (6j)

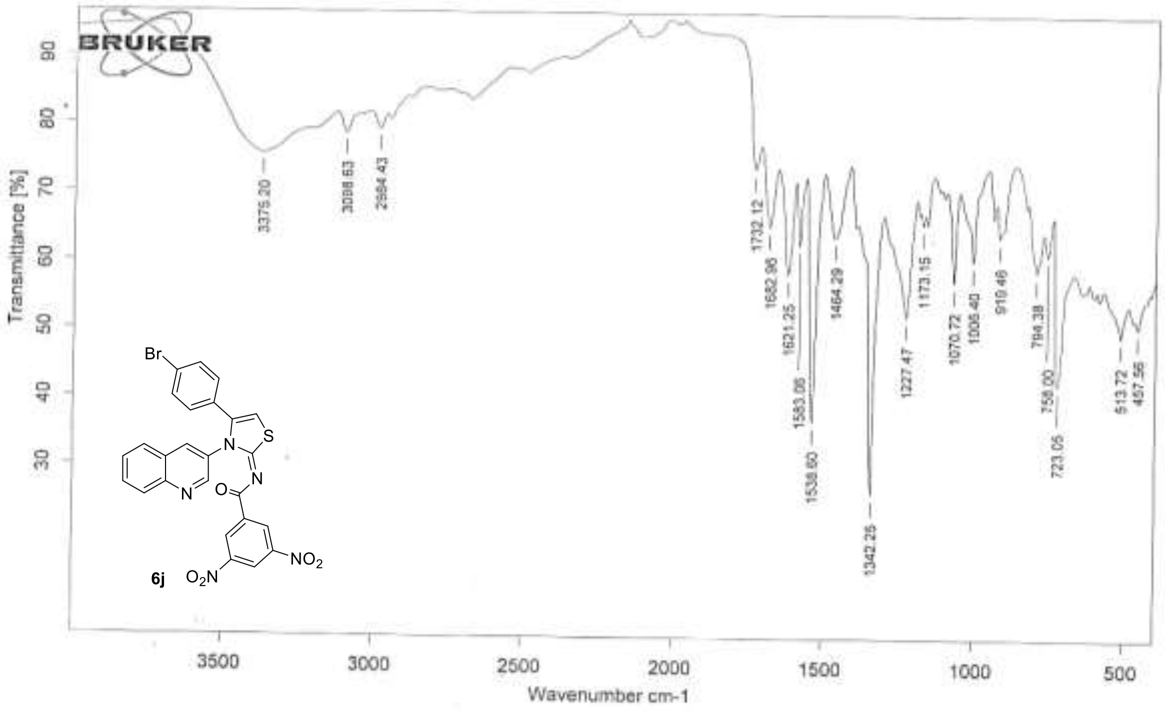

HPLC-MS

(*E*)-*N*-(4-(4-Bromophenyl)-3-(quinolin-3-yl)thiazol-2(3*H*)-ylidene)butyramide (6a)

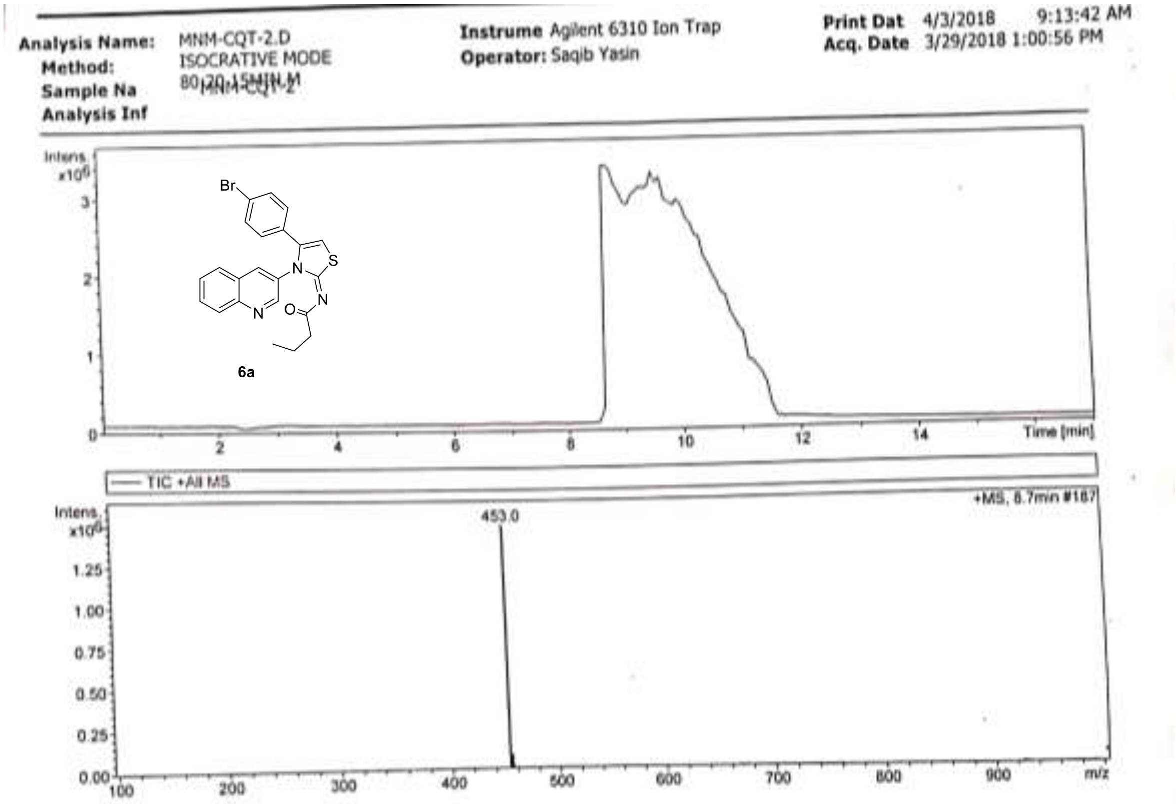

Information Classification: General

(*E*)-*N*-(4-(4-Bromophenyl)-3-(quinolin-3-yl)thiazol-2(3*H*)-ylidene)pentanamide (6b)

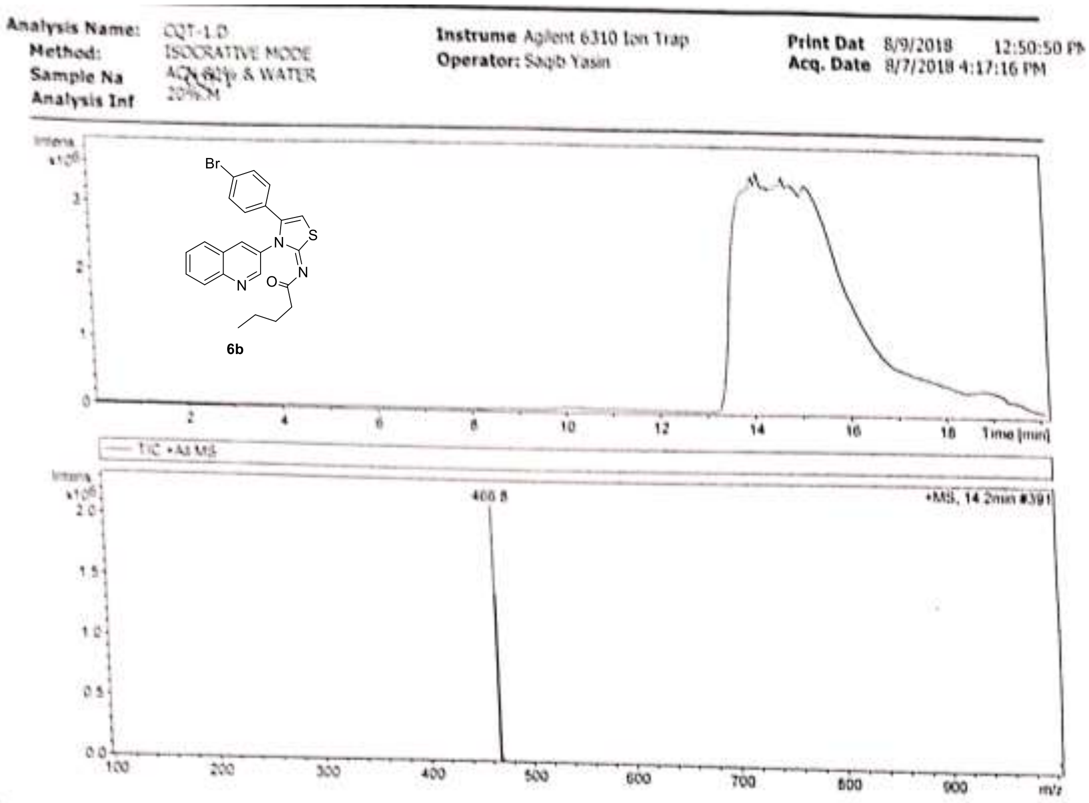

(*E*)-*N*-(4-(4-Bromophenyl)-3-(quinolin-3-yl)thiazol-2(3*H*)-ylidene)hexanamide (6c)

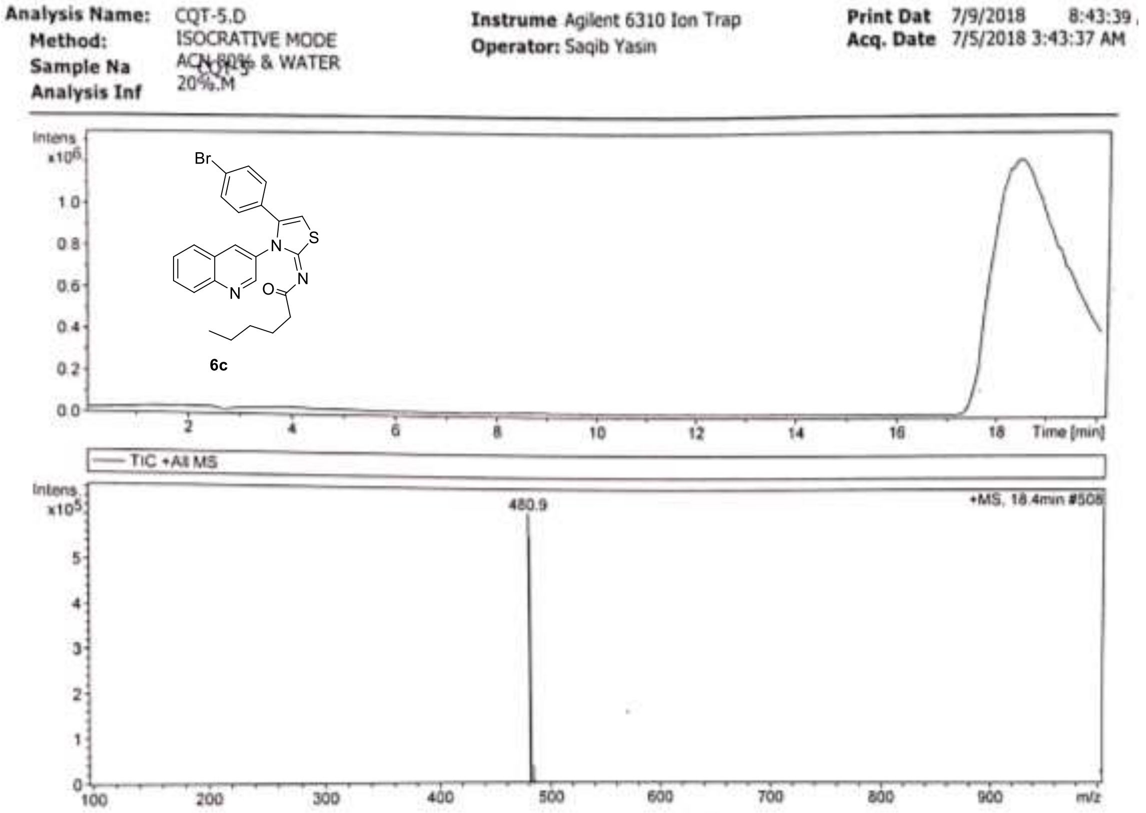

Alkaline phosphatase inhibition assay and kinetic mechanism analysis

The reaction mixture comprised of 50 mM Tris-HCl buffer (5 mM $MgCl_2$, 0.1 mM $ZnCl_2$ pH 9.5), the compound (0.1 mM with final DMSO 1% (v/v) and mixture was pre-incubated for 10 min by adding 5 µL of CIALP (0.025 U/mL). Then, 10 µL of substrate (0.5 mM *p*-NPP (para nitrophenyl phosphate disodium salt) was added to initiate the reaction and the assay mixture was incubated again for 30 min at 37 °C. The change in absorbance of released *p*-nitrophenolate was monitored at 405 nm, using a 96-well microplate reader (SpectraMax ABS, USA). All the experiments were repeated three times in a triplicate manner. KH_2PO_4 was used as the reference inhibitor of CIALP. The Alkaline Phosphatase activities were calculated according to the following formula:

$$\text{Alkaline Phosphatase activity } (\%) = (\text{ODcontrol} - \text{ODsample} \times 100) / \text{ODcontrol}$$

Where $OD_{control}$ and OD_{sample} represents the optical v densities in the absence and presence of sample, respectively.

The inhibitor **6g** concentrations were used 0.00, 0.169, 0.337 and 0.674 µM, Substrate *p*-NPP concentrations were 10, 5, 2.5, 1.25, 0.625 and 0.3125 mM. Pre-incubation time and other conditions were same as described in alkaline phosphatase inhibition assay section. Maximal initial velocities were determined from initial linear portion of absorbances up to 10 minutes after addition of enzyme at per minute's interval. The inhibition type on the enzyme was assayed by Lineweaver-Burk plot of inverse of velocities (1/V) versus inverse of substrate concentration 1/[S] mM^{-1}. The EI dissociation constant *K*i was determined by secondary plot of 1/V versus inhibitor concentration

Information Classification: General
